# Effects of Interrupting Prolonged Sitting with Physical Activity Breaks on Blood Glucose, Insulin and Triacylglycerol Measures: A Systematic Review and Meta-analysis

**DOI:** 10.1007/s40279-019-01183-w

**Published:** 2019-09-24

**Authors:** Roland Loh, Emmanuel Stamatakis, Dirk Folkerts, Judith E. Allgrove, Hannah J. Moir

**Affiliations:** 1 School of Life Sciences, Pharmacy and Chemistry, Faculty of Science, Engineering and Computing, Kingston University, Penrhyn Road, Kingston upon Thames, Surrey, London, KT1 2EE UK; 2 Charles Perkins Centre, Prevention Research Collaboration, School of Public Health, University of Sydney, Sydney, NSW Australia; 3 Faculty of Sport and Exercise Sciences, University of Muenster, Münster, Germany

## Abstract

**Background:**

Physical activity (PA) breaks in sitting time might attenuate metabolic markers relevant to the prevention of type 2 diabetes.

**Objectives:**

The primary aim of this paper was to systematically review and meta-analyse trials that compared the effects of breaking up prolonged sitting with bouts of PA throughout the day (INT) versus continuous sitting (SIT) on glucose, insulin and triacylglycerol (TAG) measures. A second aim was to compare the effects of INT versus continuous exercise (EX) on glucose, insulin and TAG measures.

**Methods:**

The review followed the Preferred Reporting Items for Systematic Reviews and Meta-analyses (PRISMA) recommendations. Eligibility criteria consisted of trials comparing INT vs. SIT or INT vs. one bout of EX before or after sitting, in participants aged 18 or above, who were classified as either metabolically healthy or impaired, but not with other major health conditions such as chronic obstructive pulmonary disease or peripheral arterial disease.

**Results:**

A total of 42 studies were included in the overall review, whereas a total of 37 studies were included in the meta-analysis. There was a standardised mean difference (SMD) of − 0.54 (95% CI − 0.70, − 0.37, *p *= 0.00001) in favour of INT compared to SIT for glucose. With respect to insulin, there was an SMD of − 0.56 (95% CI − 0.74, − 0.38, *p *= 0.00001) in favour of INT. For TAG, there was an SMD of − 0.26 (95% CI − 0.44, − 0.09, *p *= 0.002) in favour of INT. Body mass index (BMI) was associated with glucose responses (*β* = − 0.05, 95% CI − 0.09, − 0.01, *p* = 0.01), and insulin (*β* = − 0.05, 95% CI − 0.10, − 0.006, *p* = 0.03), but not TAG (*β* = 0.02, 95% CI − 0.02, 0.06, *p* = 0.37). When energy expenditure was matched, there was an SMD of − 0.26 (95% CI − 0.50, − 0.02, *p *= 0.03) in favour of INT for glucose, but no statistically significant SMDs for insulin, i.e. 0.35 (95% CI − 0.37, 1.07, *p *= 0.35), or TAG i.e. 0.08 (95% CI − 0.22, 0.37, *p *= 0.62). It is worth noting that there was possible publication bias for TAG outcomes when PA breaks were compared with sitting.

**Conclusion:**

The use of PA breaks during sitting moderately attenuated post-prandial glucose, insulin, and TAG, with greater glycaemic attenuation in people with higher BMI. There was a statistically significant small advantage for PA breaks over continuous exercise for attenuating glucose measures when exercise protocols were energy matched, but no statistically significant differences for insulin and TAG. PROSPERO Registration: CRD42017080982.

**PROSPERO Registration:**

CRD42017080982.

**Electronic supplementary material:**

The online version of this article (10.1007/s40279-019-01183-w) contains supplementary material, which is available to authorized users.

## Key Points

**Table:** 

Breaking up sitting with physical activity (PA) moderately attenuated post-prandial glucose and insulin, with a small effect size attenuation for TAG.
There was greater glycaemic attenuation in people with higher body mass index (BMI).
PA breaks were slightly more effective for glycaemic attenuation compared to one continuous bout of PA when experimental conditions were energy expenditure matched.

## Introduction

### Rationale

Increasing physical activity (PA) [1] and both decreasing and interrupting “sedentary behaviour” are emphasised in public health guidelines [2]. “Sedentary behaviour” (SB) is any seated or reclining behaviour, whilst awake, with energy expenditure (EE) at or below 1.5 metabolic equivalents (METs) [3, 4], such as sitting in the office. The UK Department of Health [2] recommends breaking up long periods of sitting during working hours and interrupting sedentary time. Australia’s Department of Health [5] recommends interrupting long sitting periods, although no quantitative threshold is specified.

A systematic review and meta-analysis of cross-sectional observational and laboratory-based experimental studies on the effects of breaks in SB [6] concluded that walking-based light-intensity physical activity (LIPA) and moderate intensity physical activity (MPA) breaks resulted in significant reductions in post-prandial glucose and insulin. Physical activity (PA) breaks in sitting were also more effective than one continuous bout of exercise on glucose. Nonetheless because this review only included five studies on glucose, published between 2011 and 2014, some relevant earlier studies [7–12] and more recent studies [13–22] might have been omitted or missed. There was no date restriction in Benatti et al. [23] but no meta-analysis was performed. Therefore, the magnitude and moderators of PA breaks on metabolic variables compared to sitting were not quantitatively assessed. It also remains to be established if PA breaks influence metabolic markers in a different way to structured continuous exercise, and thus confer a different benefit to structured continuous exercise. Recently, the United States of America Physical Activity Guidelines Advisory Committee in its Scientific Report to the Secretary of Health and Human Services stated a need for randomised controlled trials to test the effects of interventions to replace time spent in SB with PA [24]. Therefore, an updated meta-analysis of such existing trials, in adults, whether healthy or with type 2 diabetes, that can be used as part of the development of public health guidelines, is apposite.

Accordingly, there is scope for a new systematic review and meta-analysis of the experimental literature on the metabolic effects of interruptions of prolonged sitting with PA breaks, as an important contributor to the evidence pool used to develop, update, and refine public health guidance.

### Objective

The primary aim was to systematically review and meta-analyse that studied the effects of controlled trials breaking up prolonged sitting with PA breaks throughout the day compared with prolonged sitting on glucose, insulin and TAG. A secondary aim was to systematically review and meta-analyse controlled trials that compared the effects of PA breaks against continuous exercise on glucose, insulin and TAG.

## Methods

The review adhered to PRISMA recommendations [25, 26], and is registered at the International Prospective Register of Systematic Reviews (PROSPERO) (identification code: CRD42017080982).

### Search Strategy

Firstly, a systematic database search of PubMed, OvidSP, Journals@Ovid and PsycINFO, Science Direct, and SPORTDiscus, was conducted on 04/03/2017. The search was subsequently updated on 03/07/2018. Search terms were collated into four broad categories, based on the PICOT (population, intervention, comparison, outcome, time) format [26, 27]: setting (“sedentary behaviour”), intervention (“physical activity”), intervention type/comparison (“breaks”), outcomes (“glucose”) [28]. Full search details terms for all databases searched are provided in Electronic Supplementary Material Appendix S1.

Additionally, a hand search of the reference lists of articles included in the final analysis that were identified via the database search was conducted, as were the first 20 “related articles”, via the “related articles” link on PubMed, of those included database search articles. A hand search of other reviews, commentaries, letters, PhD dissertations, and reference lists of original articles was also conducted.

### Study Selection

Studies were then selected according to the following inclusion and exclusion criteria. Studies were included if they fulfilled all of the following criteria, with PICOT categories in parentheses where appropriate:

1. Participants aged 18 years or above (population).
2. Included as an outcome at least one measure of continuous glucose monitoring system or blood glucose, insulin or TAG measures, such as area under the curve (outcomes).
3. Studies with participants with type 2 diabetes (T2D), prediabetes, impaired fasting glucose (IFG) or obesity (population). Type 2 diabetics were included as the outcome variables assessed, specifically glucose and insulin are of direct relevance to type 2 diabetes. Additionally, the daily habitual PA of type 2 diabetics is not influenced by their condition.
4. Published peer reviewed prospective intervention studies, assessing explicitly breaking up sitting time with some form of physical activity (intervention), such that there would either be: (a) at least one condition in which a bout of continuous prolonged sitting (comparison) occurred, and another condition in which such sitting was intermittently broken up with multiple PA bouts spread throughout the sitting bout (intervention); or (b) one condition in which a bout of continuous prolonged sitting was broken up with multiple PA bouts spread throughout the sitting bout (intervention) and one condition in which there was a continuous bout of exercise performed during a sitting bout (intervention). One bout of continuous exercise was defined as one continuous non-stop bout of exercise without any rest periods in between. A sitting bout was defined as a bout in which continuous prolonged sitting occurred, such that participants were reported to be sitting or sedentary or rested in the laboratory.
5. The study attempted to control for/manipulating sitting and PA break conditions, with the sitting (comparison) and PA breaks protocol (intervention) was clearly reported.
6. Different conditions in cross-over trials conducted separately on different days, to minimise carryover effects (comparison).
7. Trials in which the PA breaks and sitting bouts protocol was not controlled or clearly reported were included in the narrative review, but not meta-analysed.
8. English language articles.

Studies were excluded if they met any of the following criteria:

1. Different trial conditions were performed on the same day, without a washout period.
2. If the study included an experimental condition comparing a continuous exercise bout against a sitting bout condition, but no condition in which sitting was broken up with multiple short physical activity bouts.
3. No attempt was made to control for sitting bouts, for example, if participants during an exercise trial condition were permitted to be absent from the laboratory when not exercising, or if the sitting and breaks protocol was not monitored to adhere to an explicitly reported protocol. However, such studies were included in the narrative summary, but not the meta-analysis.
4. The only intervention used to interrupt sitting was standing, as standing may have minimal impact on EE compared to sitting activities [29, 30]. Furthermore, it has been reported that inter-individual heterogeneity in EE during standing might be due to leg or body displacement, such that heterogeneity in effectiveness of standing interventions might be due to such variations [29, 30]. Additionally, normal weight men and women, BMI: 22.5 ± 1.5 kg/m^2^, had higher leg muscle activity during sitting compared to the overweight, BMI: 28.4 ± 2.9 kg/m^2^. Conversely, leg muscle activity was higher in overweight adults during standing [31]. Thus, standing studies were excluded.
5. Reused data from a previous study, without containing any new measurements for at least one of glucose, insulin or TAGs.
6. Participants were from special/clinical populations, for example patients with peripheral arterial disease or chronic obstructive pulmonary disease. Studies with participants with chronic obstructive pulmonary disease (COPD) or peripheral arterial disease (PAD) were excluded as the aim of the meta-analysis was not to assess the effects of physical activity breaks on rehabilitation, especially rehabilitation from cardiopulmonary disease or cancer.
7. Commentaries, letters, reviews, conference abstracts, poster abstracts, theses or dissertations.
8. Non-English articles.

**Fig. 1:**
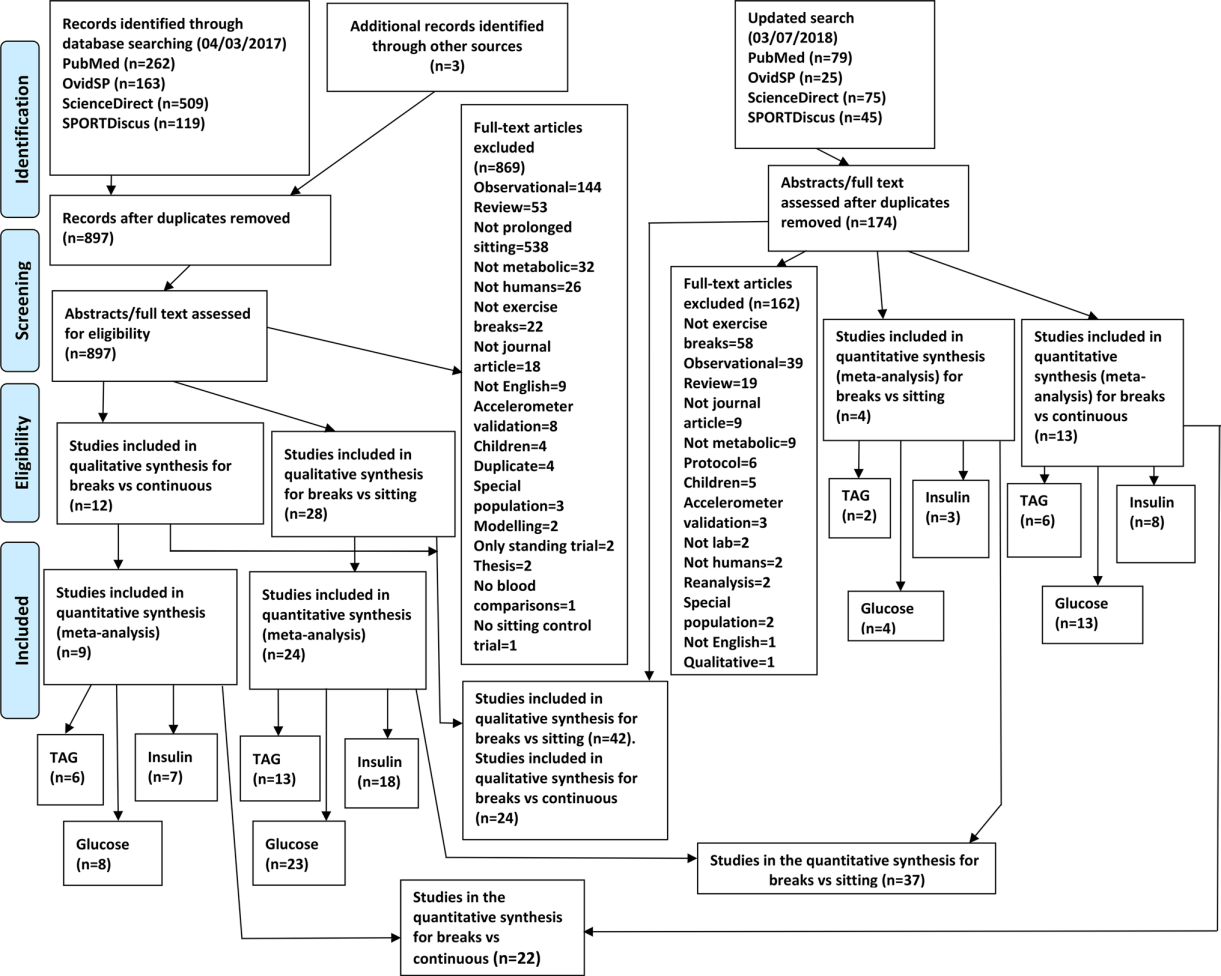
Modified PRISMA flow diagram for included and excluded studies

Studies were independently assessed for inclusion by two reviewers, RL, DF, with disagreements resolved via discussion. The reviewers, RL, DF, were not blinded to authors, institutions or journals of publication. If a decision on whether to include or exclude a paper could not be made from the title and abstract, the full text was obtained and checked. The flow diagram for the search process is presented in Fig. 1. A complete list of excluded studies, with reasons for exclusion, is available upon request.

### Data Extraction

Data from included studies were extracted (by RL) for first author name, publication date, participant characteristics, full description of the PA and sitting intervention protocol and outcomes. Outcomes extracted for the narrative review were measures of glucose, insulin, triacyglycerol, c-peptide, non-esterified fatty acids (NEFA), cholesterol, lipoproteins from blood whether plasma, serum or whole, and blood pressure.

### Risk of Bias Assessment

The Cochrane Collaboration’s risk of bias (RoB) tool [32] was used to aid in assessing the RoB in individual studies. Components were assessed independently, with no overall composite score assigned, as per PRISMA [25, 26] and Cochrane collaboration [32, 33] recommendations. Washout period for crossover studies was used for the “other” sources of bias component. Each component rated was as “high risk” or “low risk”. If details for a particular domain were insufficient, the risk of bias was assessed as “unclear”. Assessments were performed independently by two authors (RL, DF) with disagreements resolved by discussion, and then arbitration (HJM) if necessary.

### Data Synthesis

A narrative overview provided in text and tables summarises study characteristics. The narrative synthesis includes studies in which PA break or sitting protocols were not strictly controlled to provide a broader summary of the literature, whereas only controlled laboratory studies were statistically meta-analysed.

C-peptide, blood pressure, NEFA, cholesterol and lipoprotein outcomes were not meta-analysed because few studies had these variables as outcomes. Studies with glucose, insulin and TAG measures were meta-analysed. Interstitial glucose data via continuous glucose monitoring system (CGMS), if available, were extracted for the meta-analysis as a first preference over post-prandial measures of venous or capillary blood glucose, as continuous glucose data, as opposed to the snapshot nature of venous or capillary blood draw, provides a more comprehensive view of glucose responses, that is not dependent on the blood draw schedule. Incremental area under the curve (iAUC) for glucose, insulin, TAG was meta-analysed in preference to total area under the curve (tAUC), as iAUC is the recommended measure for detecting differences in post-prandial responses [34–36]. Data from prior studies that were reanalysed, combined for reanalysis, and reported in a later study were not extracted. If a later publication reported a new measure of, for example glucose, obtained from the same experimental conditions as a prior publication, CGMS glucose was used as the first preference, if available. If this was not available, post-prandial iAUC was used, followed by tAUC.

Means, standard deviations or standard errors or 95% confidence intervals (CI) were extracted from individual studies and used to calculate standardised mean differences (SMD) using DerSimonian and Laird random-effects models [33]. Continuous outcomes were analysed using SMD to account for different measurement scales [37], tAUC or iAUC over different time scales. When multiple exercise conditions were used in a study, data for all relevant conditions were synthesised and reported separately in the appropriate meta-analysis.

If a study contained more than 2 trial arms, and a control comparison condition was used twice in the meta-analysis, the sample size for the control condition was divided by the number of times the control condition was used [33]. If means were not reported, and medians were reported instead, the study was not meta-analysed. Pooled continuous data were expressed as SMD with 95% CI. SMDs were interpreted according to Cohen [38]: 0.2 represents a small effect, 0.5 a moderate effect, and 0.8 a large effect.

### Missing Data

When required outcome data for glucose, insulin and TAG were not available in the full text, but data were presented graphically, an attempt was made to digitise the graph. If this was not possible, the original authors were contacted. If data still could not be obtained successfully, the affected study was omitted from the meta-analysis, and the results summarised in the narrative review.

### Assessment of Heterogeneity

Statistical heterogeneity was tested with the Chi-square test (*p* < 0.05) and I^2^ statistic (0–40%: might not be important; 30–60%: may represent moderate heterogeneity; 50–90%: may represent substantial heterogeneity; 75–100%: considerable heterogeneity) [33].

### Subgroup Analysis

Subgroup analysis for TAG was pre-specified [33] according to whether the experimental condition was performed on 1 day, or over multiple days, as there is considerable evidence that the effects of exercise on TAG peak approximately 18 h post-exercise [39, 40]. Usual PA, body mass index, cardio-respiratory fitness (CRF) or insulin resistance status of participants was selected as another subgroup characteristic, given that metabolic responses to exercise might be affected by CRF or insulin sensitivity status [41–44], with one subgroup consisting of studies that assessed participants who were physically inactive, or sedentary, or were overweight/obese or had type 2 diabetes or impaired fasting glucose, and the other subgroup containing physically active participants. “Physically active” was defined as either exceeding the recommended 150 min of moderate to vigorous physical activity (MVPA) per week, or reported as “recreationally active” [7, 8, 45]. “Sedentary” was defined as not working in a non-sedentary job [46], exceeding 5 h of sitting time per day [15, 22, 47], or any study that defined participants as sedentary. If a study did not report the PA, body mass or health status of participants, it was omitted from the subgroup analysis. Subgroup analysis was also performed for sex, as sex might affect metabolic responses to exercise, feeding, and metabolic health [48–51], possibly due to the effects of sex hormones such as oestrogen [52]. As EE of exercise might affect results, subgroup analysis was also performed to determine whether EE between conditions was matched when comparing PA breaks with continuous exercise.

### Meta-regression

Meta-regression was only performed, to explore the possible effects of any explanatory variable on differences in post-prandial glucose, if at least ten studies were included in the meta-analysis, as there should be at least ten studies in a meta-regression for each explanatory variable modelled [33]. If there were sufficient studies, a random-effects model was used to assess whether body mass index (BMI) moderates the effect, as evaluated by SMD, of PA breaks compared with sitting, and of PA breaks vs continuous exercise.

### Publication Bias

Funnel plots, Begg and Mazumdar’s rank correlation test [53], Egger’s regression test [54] and Rosenthal’s fail-safe N [55] were used to assess publication bias if more than ten studies were included in the meta-analysis [26, 56]. The trim and fill method, with L0 as the estimator [57], was used to estimate “missing” studies, if any, in the funnel plots. The method of Vevea and Woods [58] was used to calculate the modified SMD in the event of severe 2-tailed selection bias.

### Statistical Analysis

Graphical representations of potential bias within and across studies are presented using Review Manager 5.3 (RevMan5.3) (Cochrane Collaboration, Copenhagen, Denmark). All statistical calculations for summary measures were analysed in RevMan 5.3 and presented as SMD and 95% CI. Meta-regression and publication bias analyses were performed in R (The R Project for Statistical Computing). Statistical adjustment of SMD for publication bias was performed in SPSS version 23 (IBM Corporation, Armonk, NY, USA) and R, using the macros developed by Field and Gillett [59].

## Results

### Studies Retrieved

The initial database search was performed on 04/03/2017. Subsequently, the search was updated on 03/07/2018. There were 897 studies in the initial search results after removal of duplicates. 28 studies met the inclusion criteria. In the updated search results, there were 174 studies after removal of duplicates, of which 14 met the inclusion criteria. Therefore, a total of 42 studies were included in the final systematic review, of which 37 were included in the meta-analysis. The results of the systematic search are presented in Fig. 1.

### Characteristics of Included Studies

#### PA Breaks vs No-Exercise Sitting

**Fig. 2:**
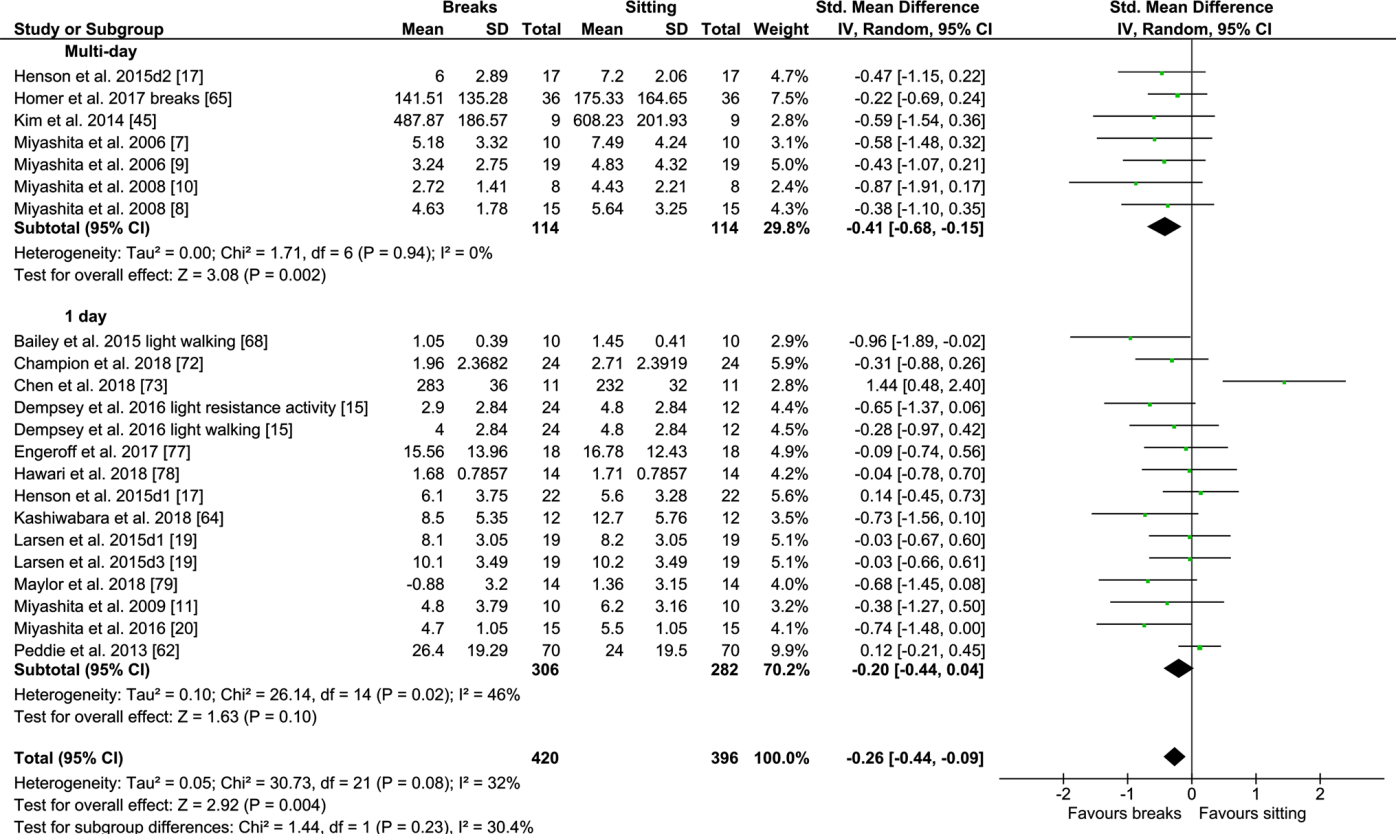
Forest plot for the effects of physical activity (PA) breaks on TAG measures, multi-day vs 1 day; D1: Day 1, d2: day 2

**Fig. 3:**
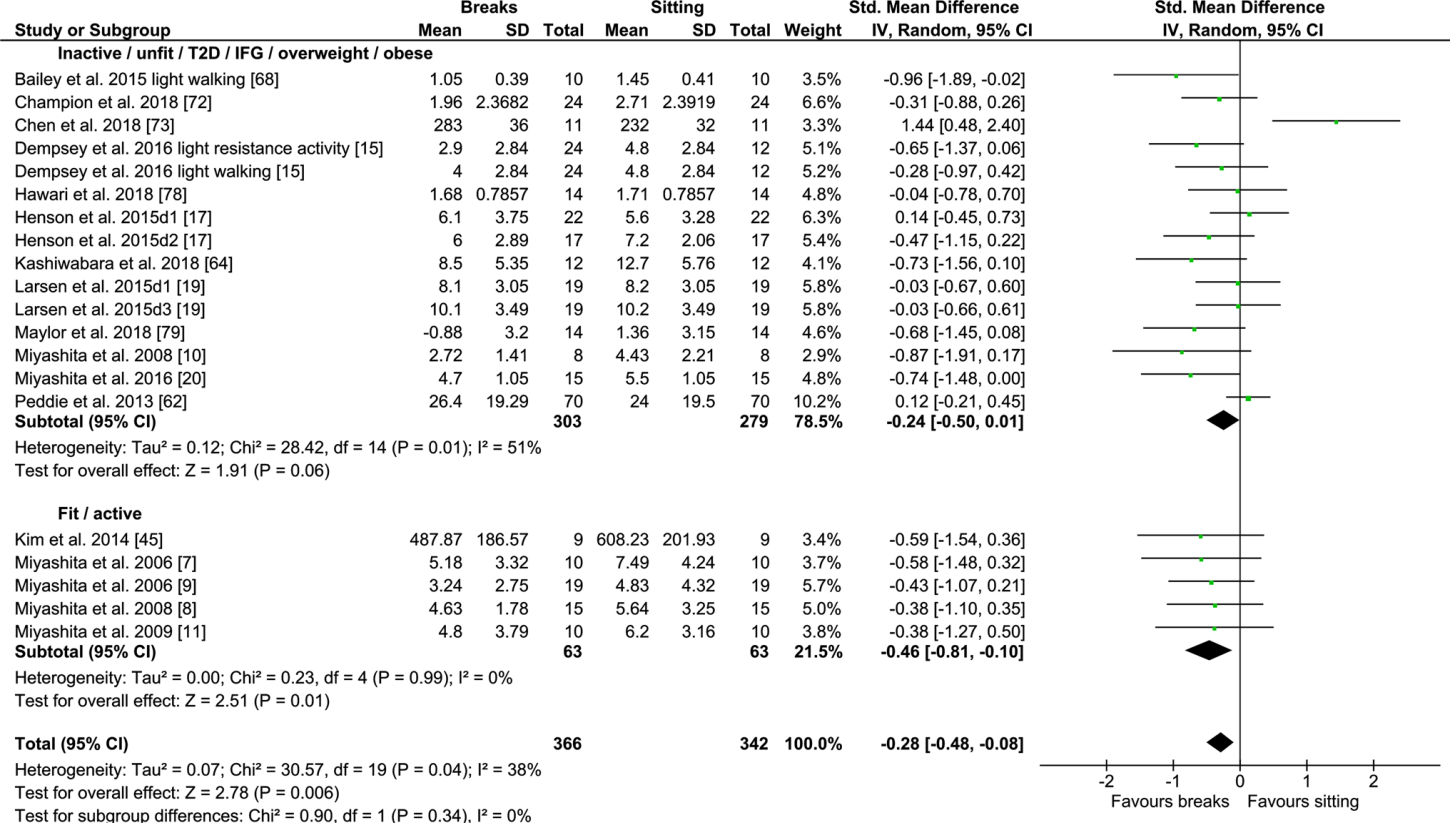
Forest plot for the effects of physical activity (PA) breaks on TAG measures. D1: day 1, D2: day2

**Fig. 4:**
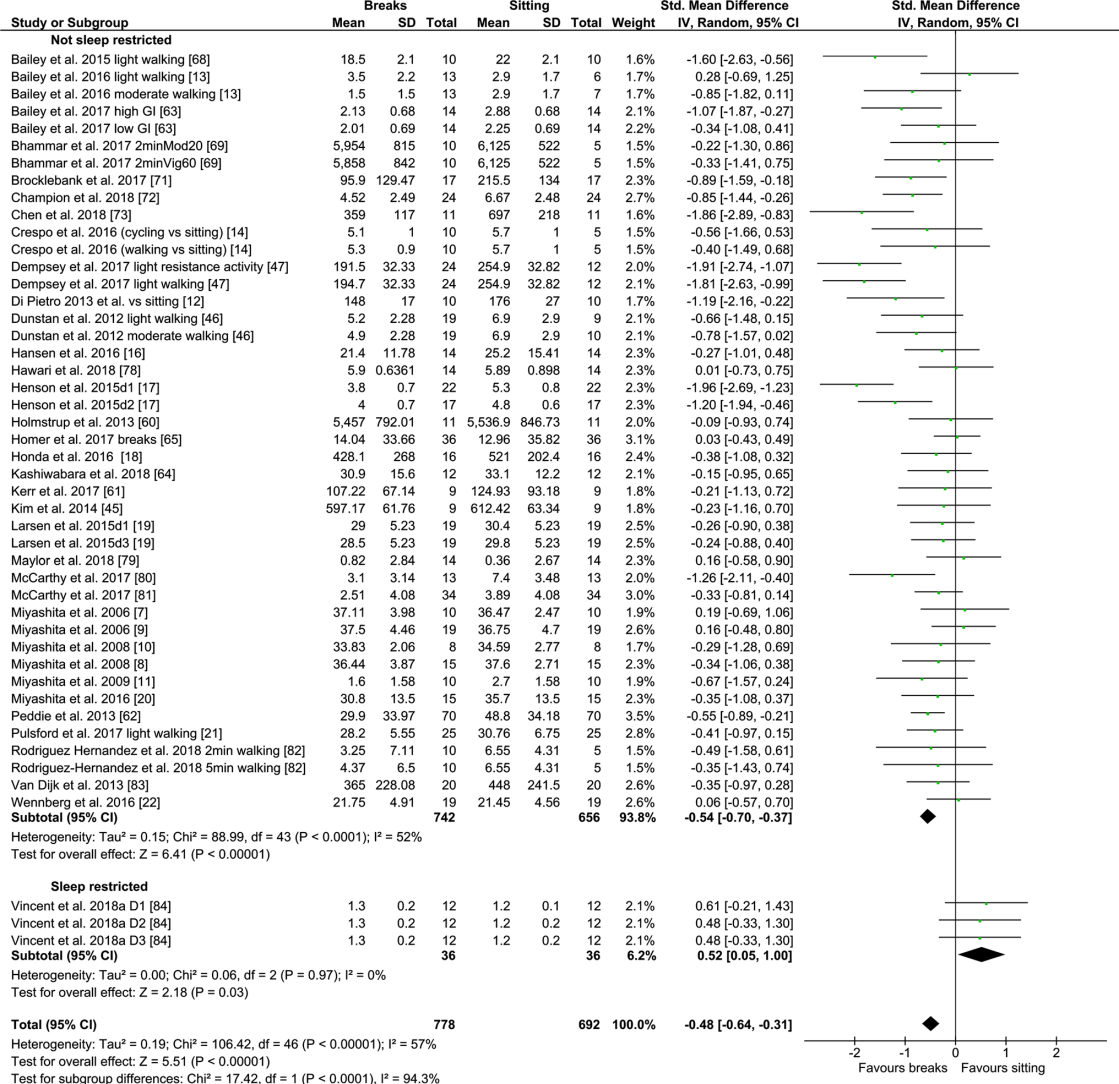
Forest plot for the effects of physical activity (PA) breaks on glucose measures; GI: glycaemic index

**Fig. 5:**
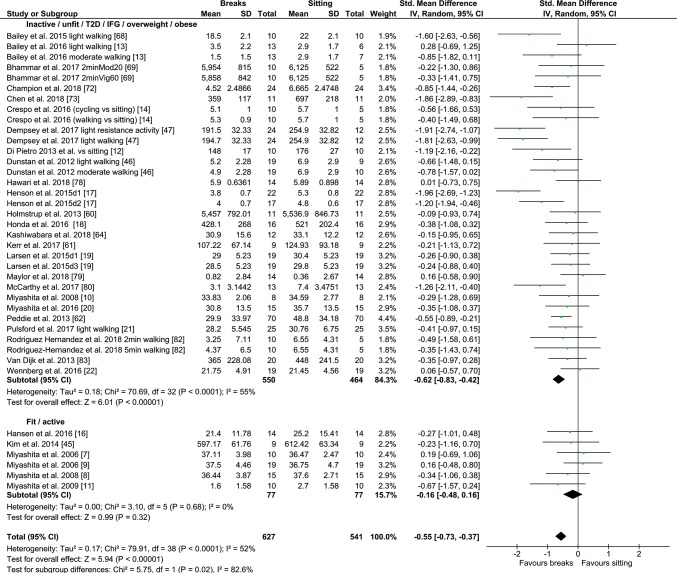
Forest plot for the effects of physical activity (PA) breaks on glucose measures, active vs inactive/unfit/T2D/IFG/overweight/obese; T2D; type 2 diabetes, IFG: impaired fasting glucose, GI: glycaemic index

**Fig. 6:**
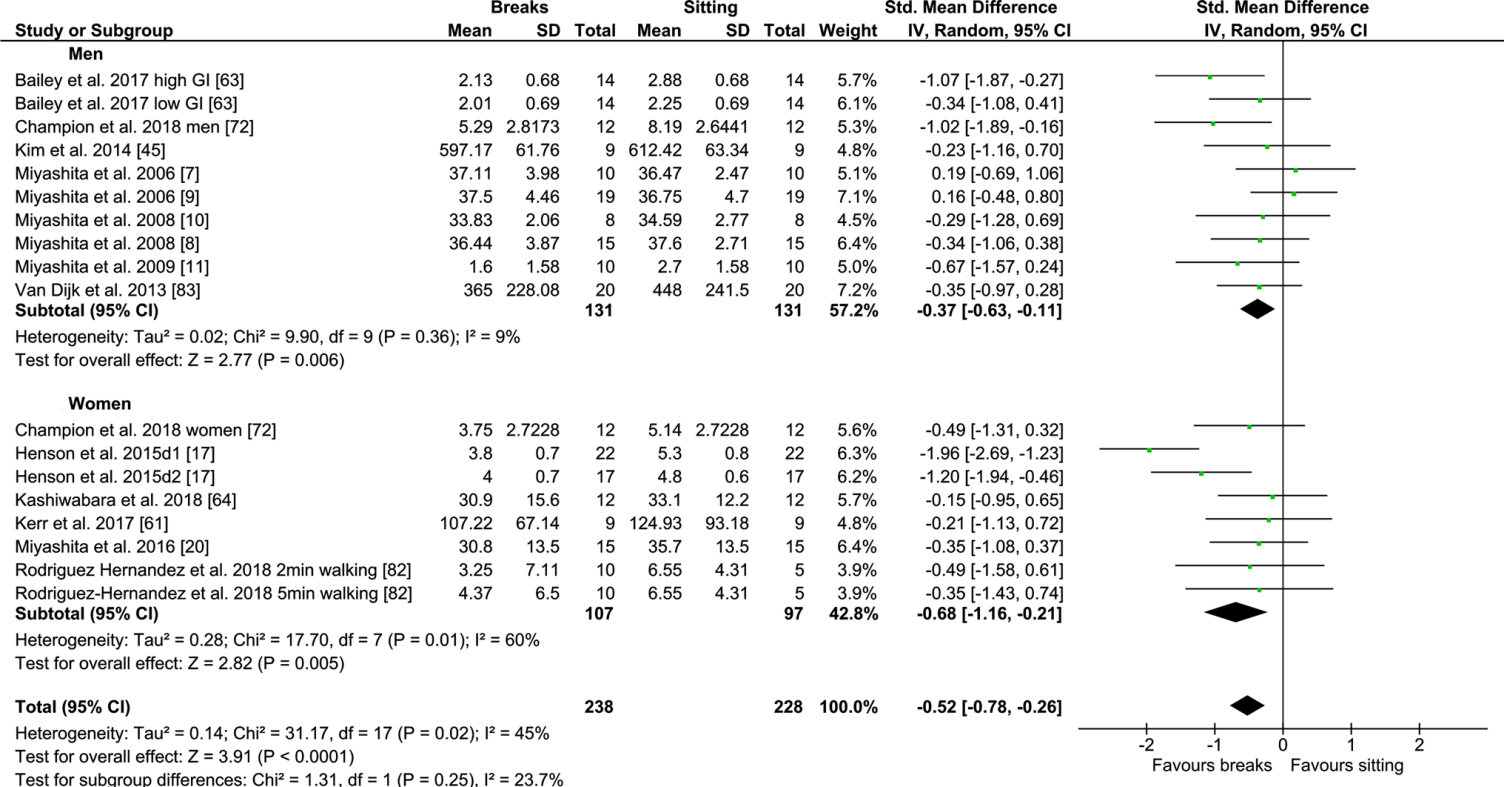
Forest plot for the effects of physical activity (PA) breaks vs continuous exercise on glucose measures, stratified by sex; GI: glycaemic index

**Fig. 7:**
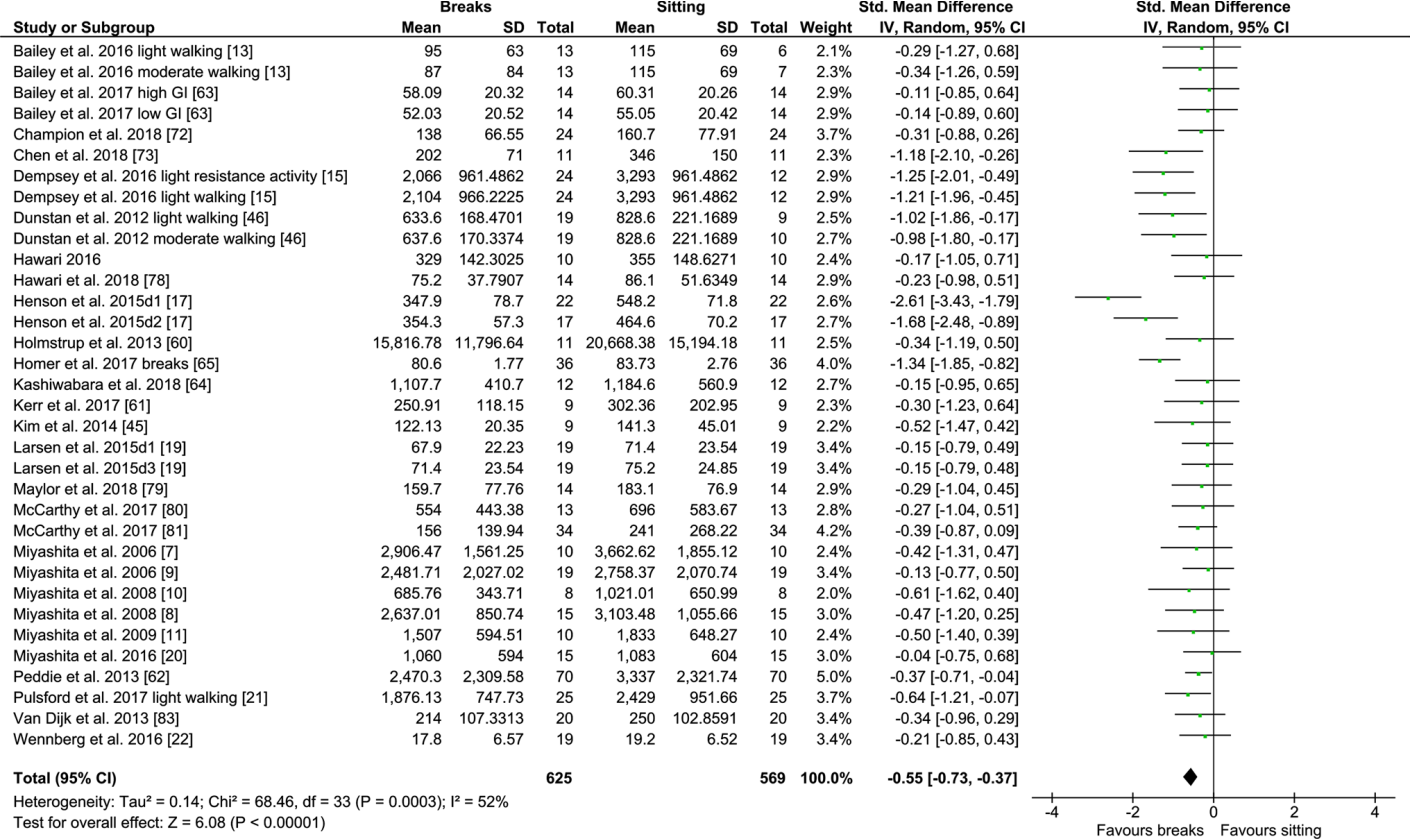
Forest plot for the effects of physical activity (PA) breaks on insulin measures; GI: glycaemic index

**Fig. 8:**
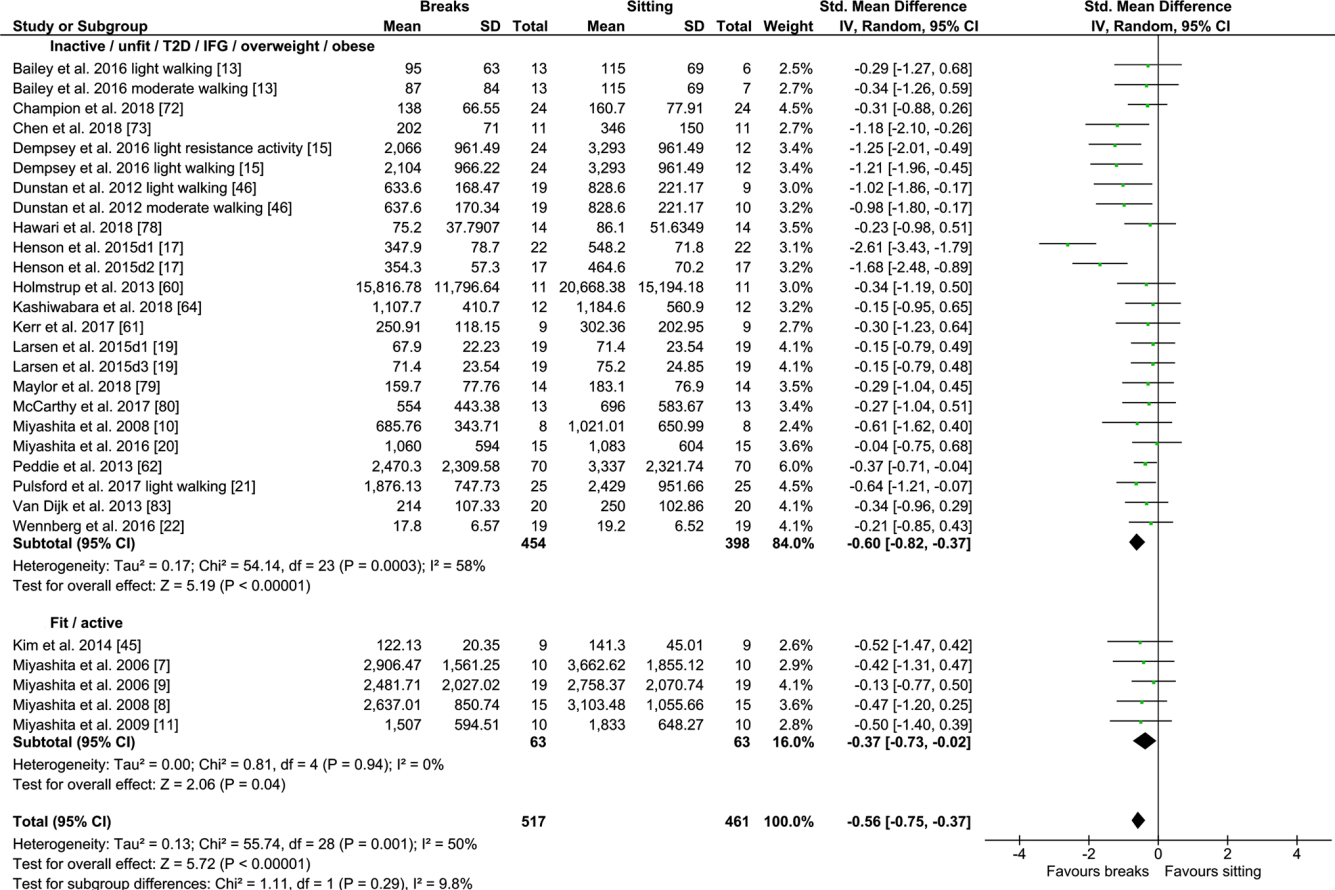
Forest plot for the effects of physical activity (PA) breaks on glucose measures, active vs inactive/unfit/T2D/IFG/overweight/obese; T2D; type 2 diabetes, IFG: impaired fasting glucose, GI: glycaemic index

In total, 42 studies were reviewed. Participants ranged from those with type 2 diabetes [15, 47, 60] to those who were healthy and had relatively high levels of CRF [7, 8, 45]. The number of participants in studies ranged from 9 [45, 61] to 70 [62]. A total number of 620 participants were included in the meta-analysis for glucose outcomes, 523 for insulin outcomes and 360 for TAG outcomes. Participants were from 22.1 [63] to 70.5 years old [64]. Most studies utilised 1 day designs, but some utilised multi-day designs [7–10, 17, 19, 45, 65]. Altenburg et al. (80) was omitted from the meta-analysis, but included in the narrative summary (Table 1) as data were skewed, and might have violated the underlying assumptions of normality of data distribution [33, 66] for the statistical models used in the meta-analyses. Forest plots for TAG outcomes are presented in Figs. 2, 3, Electronic Supplementary Material Appendix S2—Fig. S1; for glucose outcomes in Figs. 4, 5, 6; and for insulin outcomes in Figs. 7, 8, Electronic Supplementary Material Appendix S2—Fig. S2.

**Table 1:** Studies comparing PA breaks with sitting

Study	Participants	Protocol	Outcomes	Results (please see table footnotes for interpretation of results)
Altenburg et al. [67]	5 M 6 W (median, 25%tile–75%tile); age: 21.4 y (19.5–23.1); BMI: 23.2 kg/m^2^; PA/SB unmentioned	SIT: 1 h baseline + 7 h sitting; INT: sitting (372 min) + 8 min cycling @ 40–60% (52 ± 3.2%) HRR, RPE: 11.2 ± 1.6, hourly (6 × 8) (1st session @ 0 h + 1)	Capillary @ baseline, hourly before exercise: C-peptide, glucose, TAG, HDL-C, LDL-C, TC	C-peptide: INT < SIT; TAG, TC, HDL-C, LDL-C, glucose: ↔
Bailey and Locke [68]	7 M 3 W (mean ± SE); age: 24 ± 3 y; BMI: 26.5 kg/m^2^ ± 4.3; healthy, PA unmentioned	SIT: sitting 5 h; STAND: sitting (272 min) +2 min standing every 30 min; WALK: sitting (272 min) +2 min walking (3.2 km/h) every 30 min	Capillary: baseline, hourly, before exercise: glucose, BP baseline and 5 h for TAG, HDL-C, TC	Glucose: WALK < STAND and SIT
Bailey et al. [13]	Healthy 6 M 7 W (mean ± SD); age: 26.6 ± 8 y, < 150 min/w MVPA), not in non-sedentary job; BF: 24.4% ± 8.2%	SIT: sitting 5 h; LIGHT: sitting (272 min) +2 min walking (3.2 km/h) every 30 min; MOD: sitting (272 min) +2 min walking (5.8–7.9 km/h) every 30 min	Cannula: − 1 h, 0 h, hourly, before exercise: subjective appetite, acylated ghrelin, peptide YY, insulin, glucose	^a^; Glucose iAUC: men < women in CON; in men, glucose iAUC: MOD < LIGHT, MOD < CON
Bailey et al. [63]	14 M (mean ± SD); age: 22.1 ± 1.2 y, BMI: 25.0 ± 3.1 kg/m^2^, BF: 17.2 ± 5.5%	SIT + HIGH GI: high GI breakfast + 4 h sitting; SIT + LOW GI: low GI breakfast + 4 h sitting; INT + HIGH GI: high GI breakfast + 2 min walking/20 min (6.5–8.0 km/h, RPE: 12–14); INT + low GI breakfast: high GI breakfast + 2 min moderate walking/20 min (6.5 to 8.0 km/h, RPE: 12–14)	Capillary for glucose: − 15 min, 15, 30, 45, 60, 90, 120, 180, 240 min for glucose; venous: 60 min 120, 180, 240 min for insulin	^a^
Bhammar et al. [69]	5 M 5 W (mean ± SD); age: M: 31 ± 5 y W: 32 ± 6 y; BMI: M: 30.1 ± 2.3 kg/m^2^, W: 30.5 ± 6.6 kg/m^2^; *V*O_2max_: M: 34.9 ± 4.0 ml/kg/min, W: 22.8 ± 2.7 ml/kg/min	SIT: 9 h sitting; 2 minMod20: 2min walking at 53 ± 5% HR_max_/3 miles/h every 20 min, total 42 min, 240 kcal. 2 minVig60: 2min walking at 79 ± 4% HR_max_ every hour, total 16 min, 140 kcal; EX: 30 min walking at 71 ± 4% HR_max_/56% *V*O_2max_/3.3 miles/h, 230 kcal	CGMS. ABP, MAP	^a^, Systolic ABP, MAP: EX < SIT
Blankenship et al. [70]	2 M 8 W (mean ± SE); age: 51.9 ± 15.4 y; BMI: 31.6 ± 10.0 kg/m^2^, BF: 42.6 ± 3.3%	EX: 30 min brisk walking, ~ 300 kcal before lunch. FLB: isoenergetic with EX, bouts of sitting ≤ 20 min; FSB: bouts of sitting ≤ 20 min, same number of breaks as FLB but time walking standing reduced to minimise EE	CGMS, catheter for blood, after MMTT at end of day, @ 30, 60, 90, 120 min, for glucose, insulin	Post-prandial glucose and insulin AUC: ↔ between conditions; glycaemic variability: FLB < EX; nocturnal hyperglycaemia: FLB < EX and FSB
Brocklebank et al. [71]	8 M 9 W (mean ± SD); age: 52.4 ± 5.1 y; BMI: 28.0 ± 4.5 kg/m^2^; 8 active, 9 inactive	SIT: 5 h sitting; WALK: 2 min corridor walking @ RPE 9 every 20 min, total 28 min	CGMS	^a^
Champion et al. [72]	12 M 12 W (mean ± SD); age: M: 32.0 ± 10.5 y, W: 39.5 ± 10.3 y; BMI: M: 26.6 ± 4.5 kg/m^2^, W: 24.8 ± 5.13 kg/m^2^; sitting time: M: 9.4 ± 2.4 h,W: 9.2 ± 2.4 h	SIT: 6 h 30 min sitting; INT: 20 min walking at 20 min, 80 min, 140 min, 200 min, 260 min, 320 min, self-selected @ 1.2–3.5 km/h, RPE 6–9	Capillary: 0 h, 45 min, 105 min, 165 min, 225 min, 285 min, 345 min, 390 min; SBP, DBP	^a^; SBP, DBP: INT < SIT
Chen et al. [73]	7 M 4 W (mean ± SD); age: 50 ± 5 y; BMI: 32.5 ± 6.7 kg/m^2^;bodyfat %: 35 ± 6%	SIT: 315 min sitting; INT, 2 min walking @ 6.4 km/h every 20 min over 315 min, 30 min total	Cannula: 0 h, hourly, and every 15 min after each meal (meal @ 0 h and 180 min), for TAG, glucose, insulin	^a^
Crespo et al. [14]	2 M 7 W (mean ± SD); age: 30 ± 15 y; BMI: 29 ± 3 kg/m^2^; 2 participants impaired fasting glucose (5.6–6.9 mmol·L^−1^), 7 prehypertensive (> 120 mmHg SBP or > 80 mmHg DBP; < 150 min/w MVPA	SIT: 8 h sitting, restroom @ 0850 h, between 1000 and 1030 h, lunch (1200–1230 h), and between 1400 and 1500 h, replicated in all conditions; Stand: 2.5 h total standing time, stand 10 min at 0850 and 0950 h, 15 min at 1045 and 1145 h, 20 min at 1240 and 1320 h, and 30 min at 1400 and 1530 h; Walk: walk @ 1mph, same frequency and duration as Stand; Cycle: ~ 20 W, 25–30 RPM, same frequency, duration as Stand	24 h CGMS, HR, activPAL	24 h glucose: Stand, Walk, Cycle < Sit, Cycle < Walk < Stand; mean glucose LAB: Cycle < Stand, EVE: Cycle < Stand and Walk, Sleep: Cycle < Sit, Stand, Walk; 6 h postprandial glucose: Cycle, Walk, Stand < Sit; Cycle < Walk < Stand; cumulative 6 h iAUC: Cycle and Walk < Sit, Cycle < Stand
Dempsey et al. (2016, 2017) [15, 47]	T2D (ADA criteria) 14 M 10 W (mean ± SE); age: 62 ± 6 y, BMI: 33.0 ± 3.4 kg/m^2^, ≥ 25 < 40 kg/m^2^;); inactive (sitting ≥ 5 h/d OR < 150 min MVPA/w for 3 months)	SIT: 7 h sitting; WALK: sitting + 3 min walking (3.2 km/h) every 30 min (12 × 3), except during lunch; SRA: sitting + 3 min calisthenics/30 min, 12 × 3 (each 3 min divided into 9 20 s segments, alternating halfsquats, calf raises, gluteal contractions, knee raises); RPE intensity (9 ± 0.3 (7–12) and 10 ± 0.3 (7–13), and HR (mean differences for HR for LW and SRA: 17 ± 1.2 bpm (8–31) and 19 ± 1.0 bpm (10–30)	Cannula: − 1 h, 0 h, then @ 30 min intervals, immediately prior to activity, for glucose, insulin, TAG, c-peptides; CGMS	Glucose: 18 h iAUC: ^a^, greater decrease for women than men for WALK and RA vs SIT; insulin: ^a^; c-peptide: WALK < SIT, SRA < SIT; TAG: SRA < SIT, SRA < WALK; EE: SRA increase of 121 ± 7% vs sitting, LW increase of 73 ± 5% vs sitting; SRA increase of 0.58 ± 0.06 kcal · min^−1^ vs LW
Di Pietro [12]	10 (mean ± SD); age: 69 ± 6 y; BMI: BMI 30 ± 5 kg/m^2^; impaired fasting glucose	INT: D1: inactive; D2: treadmill walking 3 × 15 min 3 METS postmeals; EXam: D1: inactive; D2: 45 min walking @ 3 METs @ 10.30am; EXpm: D1: inactive; D2: 45 min walking @ 3 METs @ 430 pm	CGMS, glucose; insulin only on sitting days	Glucose: INT: d2 < d1; EXAM: d2 < d1; EXPM: ↔
Dunstan et al. [46]	11 M 8 W (mean ± SD); age: 53.8 y ± 4.9 y; BMI: 31.2 kg/m^2^ ± 4.1; self-reported sedentary (sitting time > 5 h/d), < 150 min MVPA/w	SIT: sitting 7 h; LIGHT: sitting (402 min) + 2 min walking (3.2 km/h) every 20 min for 5 h; MOD: sitting (402 min) + 2 min MVPA walking (5.8–6.4 km/h) (RPE: 12–14) every 20 min	Catheter: − 2 h, − 1 h, 0 h, then hourly, before activity for glucose, insulin	^a^
Duvivier et al. [74]	2 M 16 W (mean ± SD); age: 21 ± 2 y; BMI: 22.6 ± 3.6 kg/m^2^; FPG: 4.61 ± 0.31 mmol/L	Over 4 days; SIT: 14 h sitting + 1 h walking + 1 h standing; EX: 13 h sitting + 1 h walking + 1 h standing + 1 h MVPA cycling; INT: 8 h sitting + 5 h walking + 3 h standing	Next day (day 5) fasting glucose, insulin, TAG, HDL-C, non-HDL-C, LDL-C, Apo-A, Apo-B; next day OGTT for IS	Glucose AUC/fasting: ↔; Insulin AUC: INT < SIT, INT < EX; fasting TAG: INT < SIT; fasting non-HDL-C: INT < SIT; Apo B: INT < SIT
Duvivier et al. [75]	13 M 6 W; (mean ± SD;)age: 63 ± 9 y, T2D (not on insulin), BMI: 30.5 ± 3.3 kg/m^2^, self-report MVPA: < 2.5 h/w, FPG: < 11 mmol/L	Over 4 days; SIT: 14 h sitting + 1 h walking + 1 h standing; EX: 13 h sitting + 1 h walking + 1 h standing + 1 h MVPA cycling (3 × 20 min bouts, 5 min rest between bouts); INT: 9 h sitting + 3 h walking + 4 h standing, after every 30 min sitting	Next day (day 5) 24 h CGM glucose; next day glucose, insulin fasting TAG, HDL-C, non-HDL-C, LDL-C, Apo-A, Apo-B	24 h iAUC GLUC: INT < SIT; Insulin: INT < SIT; HOMA2-IR: INT < SIT and EX; TG: INT and EX < SIT; C-peptide: INT < SIT; NEFA: SIT < INT and EX
Duvivier et al. [76]	13 M 11 W (mean ± SD); age: 64 ± 7 y, BMI: 29 ± 2 kg/m^2^, self-report MVPA: < 2.5 h/w, FPG: < 6.9 mmol/L	Over 4 days; SIT: walking and standing < 1 h/d; SitLess: ≥ 4 h/d of self-perceived light intensity walking, ≥ 3 h/d of standing, interrupt sitting every 30 min with standing/walking bouts	OGTT, catheter: 0 h, 15 min, 30 min, 45 min, 60 min, 90 min, 120 min, 190 min, for glucose, insulin, c-peptide, AG, total cholesterol, HDL-C, LDL-C, non-HDL-C, FFA, APo A-I, Apo B-100	Glucose AUC/fasting: ↔; insulin AUC/fasting: SitLess < SIT; c-peptide: AUC/fasting: SitLess < SIT; Apo B-100: SitLess < SIT; DBP: SitLess < SIT
Engeroff et al. [77]	Healthy, 18 W (mean ± SD); age: 25.6 ± 2.6 y, BMI: 21.5 ± 2.0 kg/m^2^*V*O_2max_: 41.3 ± 4.2 ml/kg/min; PA unreported	SIT: 4 h sitting; EX: 30 min cycling @ 70% VO2 max + 4 h sitting; INT: (40 min sitting + 6 min cycling @ 70% *V*O_2max_ + 40 min sitting	Venous TAG, TC, HDL-C, LDL-C, baseline, post 240 min	TAG: ↔ between conditions, overall time effect, **↑** for INT, SIT; TC: INT < EX; HDL-C: INT < EX, INT < SIT; LDL-C: ↔, **↑** for INT
Hansen et al. [16]	6 M 8 W (mean, 95%CI): age: 22 y (20–23); BMI: 23.0 kg/m^2^ (21.6–24.4); *V*O_2max_: 38.9 ml/min/kg (34.6–43.2); physically active (measured via IPAQ): 1895 MET min/W (44–3747) sedentary time: 429 (312–546)	SIT: 2.5 h sitting; INT: 2.5 h sitting interrupted with 2 min low intensity walking every 20 min (7 × 2)	Capillary: twice @ baseline, every 10 min for next 2.5 h, for glucose	↔
Hawari et al. [78]	11 M 3 W (mean ± SD); age: 37 ± 16 y, BMI: 30.5 ± 3.8 kg/m^2^	SIT: 390 min sitting; INT: 390 min sitting + 10 chair squats every 20 min over a 3 s period	Cannula: 0 h, 30 min, 60 min, 120 min, 180 min, 210 min, 240 min, 270 min, 330 min, 390 min, for glucose, insulin, TAG	^a^, Insulin: INT < SIT
Henson et al. [17]	22 W (mean ± SD); age: 66.6 ± 4.7 y; BMI: 32.9 ± 4.7 kg/m^2^; post-menopausal (> 12 m); dysglycaemic IGT (≥ 7.8 mmol/L < 11.1 mmol/L OGTT); sedentary (objectively measured < 150 min/w MVPA)	SIT: D1: 7.5 h sitting, D2: 7.5 h sitting; STAND: 6.5 sitting +5 min standing every 30 min (12 × 5) on D1, + D2 sitting; WALK: 6.5 sitting + 5 min walking, self-selected light intensity (10–12 RPE, < 4 km/h) (12 × 5) + D2 sitting	Cannula: − 1 h, 0 h, post-breakfast and lunch: 30 min, 60 min, 120 min, 180 min, for Glucose, TAG, NEFA	^a^, Glucose: STAND < SIT; NEFA: WALK > SIT, STAND > SIT
Holmstrup et al. [60]	Obese, IFG, 8 M 3 W(mean ± SE); age: 25 ± 2.6 y, BMI: 34 kg/m^2^, Men *V*O_2max_: 32.6 ± 2.5 ml/kg/min, Women *V*O_2max_: 25.5 ± 1.8 ml/kg/min; light/moderate walking ≤ 5 × /w (questionnaire)	SIT: sitting: 12 h; EX: 1 h treadmill running @ 60–65% *V*O_2peak_, after baseline blood draw and 1st meal, sitting 11 h; INT 12 × 5 mins of treadmill running @ 60–65% *V*O_2peak_ every 1 h, 1st bout after baseline blood draw and 1st meal	Catheter, baseline, every 10 min over 12 h, for glucose, insulin, c-peptides	^a^; C-peptide: EX < SIT and INT during exercise, 2 h iAUC: EX and INT < SIT
Homer et al. [65]	11 M, 25 W (mean ± SD); age: 25 (range: 19–34); BMI: 23.78 ± 4.01 kg/m^2^, *V*O_2max_: 36.19 ± mL/kg/min; Sedentary, < 150 min MVPA/w	SIT: D1: 7 h sitting, D2: 5 h sitting; EX: D1: 6 h 30 min sitting + 30 min walking @ 60% *V*O_2max_, D2: 5 h sitting; INT: D1 and D2: sitting + 2 min walking @ 60% *V*O_2max_ every 30 min; EX + INT: D1 and D2: sitting + 2 min walking @ 60% *V*O_2max_ every 30 min + 30 min walking @ 60% on D1	Cannula: D1: 0 h. D2: hourly + 30 min and 45 min post-meal, for TAG, glucose, insulin, NEFA	^a^; NEFA: ↔
Honda et al. [18]	13 M 3 W (mean ± SE); age: 65.4 ± 1.1 y, BMI: 23.6 ± 0.7 kg/m^2^, T2D	SIT: 180 min sitting; INT: 180 min sitting + 3 min stair climbing (21 steps × 6 times up and down, 80–110 steps/min) at 60 min and 120 min	Capillary: 0 min, 60, 90, 120, 150, 180 for glucose, C peptide, NEFA, lactate	Glucose: INT < CON; C-peptide: ↔; NEFA: ↔
Kashiwabara et al. [64]	12 W (mean ± SD); age: 70.5 ± 4.6 y; BMI: 25.3 ± 3.5 kg/m^2^; BP: 144 ± 19 mmHG; DBP: 85 ± 11 mmHG; inactive, < 150 min MVPA/w	SIT: 8 h sitting; INT: sitting + 1.5 min walking every 15 min @ 3.6 km/h, RPE: 11 @ 1 h, 1 h 15 min, 1 h 30 min, 1 h 45 min, 2 h 15 min, 2 h 30 min, 2 h 45 min, 4 h 15 min, 4 h 30 min, 4 h 45 min, 5 h, 5 h 15 min, 5 h 30 min, 5 h 45 min, 6 h 15 min, 6 h 30 min, 6 h 45 min, 7 h, 7 h 15 min, 7 h 30 min	Venepuncture: 0 h, 2 h, 4 h, 6 h, 8 h, for glucose, insulin, TAG, NEFA, APoB-48, ApoB-100, LPL	^a^; Apo B-48, Apo B-100, LPL: ↔
Kerr et al. [61]	9 W (mean ± SD); age: 66 ± 9 y; BMI: 30.6 ± 4.2 kg/m^2^; SBP: 123 ± 8 mmHG; DBP: 66 ± 7 mmHG	SIT: 5 h sitting; INT: 2 min walking every hour	Cannula: − 0.5 h, 0 h, every 30 min, for glucose, insulin; HR, BP, − 1 h, 0 h, every 30 min	^a^, SBP, DBP, HR: ↔
Kim et al. [45]	9 M (mean ± SD); age: 24.0 ± 4.0 y; *V*O_2max_: 51.6 ± 6.3 mL/kg/min; BMI < 30 kg/m^2^, recreationally active, healthy	SIT: D1 and D2: (7000–7500 steps/day, D3: 9 h sitting (< 2000 steps, 0900–1800), D4: HFTT; MOD: D1, D2, D4: same as SIT, D3: sitting +1 h running @ 65% *V*O_2max_ 3. INT: sitting + isoenergetic (with condition 2) intermittent walking, every hour, 9 sessions, 1st session 30 min, last session 60 min, 7 other sessions 17.8 ± 4.0 min) @ 25% *V*O_2max_ (total time: 214.5 min ± 28.0)	D4 fasting and postprandial FFA, TAG, glucose, insulin, indirect calorimetry for postprandial substrate oxidisation	^a^; FFA: MOD > INT and SIT
Larsen et al. [19]	11 M 8 W (mean ± SE); age: 56.7 ± 1.5 y; BMI: 32.7 ± 1 kg/m^2^; Sedentary (sitting > 5 h/day, self-report, < 150 min/w MVPA)	SIT: 7 h sitting; INT: sitting (402 min) + 2 min walking (3.2 km/h) every 20 min for 5 h, 3 day protocol: on D1 and D3, SIT vs INT	Cannula: − 1 h, 0 h, hourly, before exercise for glucose, insulin, TAG; model of insulin sensitivity	^a^
Maylor et al. [79]	7 M 7 W (mean ± SD); age: 29 ± 9 y, BMI: 26.1 ± 5.8 kg/m^2^, *V*O_2max_: 38.6 ± 4.2 mL/kg/min; Sedentary, inactive	SIT: 8H sitting; EX: 30 min sitting + 30 min treadmill running @ 60% VO2 reserve + 7 h sitting; INT: 30 min sitting + 2 min 32 s running @ 85% *V*O_2_ reserve every 60 min, 8 bouts	Cannula: 0 h. hourly intervals, for TAG, glucose, insulin, HDL-C	^a^; HDL-C: INT < SIT
McCarthy et al. [80]	6 M 7 W (mean ± SD); age: 66 ± 6 y; BMI: 33.8 ± 3.8;SBP: 140 ± 13 mmHG; DBP: 79 ± 9 mmHG; < 150 min MVPA/w	SIT: 7.5 h sitting; INT: 7.5 sitting + 5 min arm ergometry @ intensity similar to 3 km/h walking, total 1 h (12 ×)	Cannula: 0 h, 30 min, 60 min, 120 min, 180 min, for glucose, insulin	^a^
McCarthy et al. [81]	16 M 18 W (median ± IQR); age: M: 35 ± 17, W: 43 ± 13; BMI: M: 25.9 ± 5.1, W: 22.7 ± 4.6; *V*O_2max_: M: 50.3 ± 19.6 W: 34.0 ± 7.9; sitting: M: 547 ± 164 min, W: 595 ± 126 min	SIT: 7.5 h sitting; INT: 6.5 h sitting +5 min walking @ 3 km/h every 30 min, total 1 h	Cannula: − 1 h, 0 h, 30 min, 1 h, 2 h, 3 h, 210 min, 4 h, 5 h, 6 h, 390 min, for glucose, insulin	^a^
Miyashita et al. [7]	10 M (mean ± SE); age: 25.0 ± 1.3 y, BMI: 25.4 ± 1.2 kg/m^2^, WC: 87.2 ± 3.5 cm, BF: 9.4 ± 0.7% *V*O_2max_: 56.3 ± 1.8 mL/kg/min; Healthy, recreationally active	1.SIT: D1: 7 h sitting, D2: 7 h sitting; 2. EX: D1: 6 h 30 min sitting + 30 min running @ 71.1 ± 2.3% *V*O_2max_; D2: 7 h sitting; 3: INT: D1: 10 × 3 min running @ 69.6 ± 1.0% *V*O_2max_ between every 30 min of sitting over 7 h, D2: 7 h sitting	D2 cannula: 0 h, hourly intervals, and @ 0.5, 0.75, 3.5, 3.75 h fasting and post-prandial for TAG, glucose, insulin, NEFA, 3-OHB;	^a^; NEFA, 3-OHB: ↔
Miyashita et al. [9]	19 M (M ± SE); age: 22.7 ± 0.5 y, BMI: 23.8 ± 0.8 kg/m^2^; *V*O_2max_: 60.3 ± 2.0 mL · kg^−1^ · min^−1^	SIT: D1: sitting, 830/900 to 1600/1700, D2: 7 h sitting; INT: day1: sitting similar to SIT + 6 min running @ 70%*V*O_2max_ and 30 min rest between each running bout	D2 venous: hourly, and at 0.5 h, 0.75 h, 3.5 h, 3.75 h, for glucose, insulin, TAG, NEFA	^a^ NEFA: INT > SIT
Miyashita et al. [8]	15 M; (mean ± SE): age: 23.4 ± 0.8 y, *V*O_2max_: 56.3 ± 2.1 mL/kg/min, BMI: 23.4 ± 0.6 kg/m^2^, WC: 80.8 ± 2.1 cm, BF: 11.2 ± 0.9%, SBP: 114 ± 2 mm Hg, DBP: 68 ± 2 mm Hg; non-smoking, BP < 140/90 mmHg	1. SIT: D1: 7 h sitting, D2: 7 h sitting; 2: day1: EX: 30 min walking @ 6.8 km/h ± 0.1 (42.4 ± 1.8% *V*O_2max_) after 6 h 30 min sitting, day2: 7 h sitting; 3: INT: D1: 10 × 3 min walking 6.8 km/h ± 0.1 (41.4 ± 1.8% *V*O_2max_) between every 30 min of sitting over 7 h, D2: 7 h sitting	Next day TAG, glucose, insulin; BP: day1: baseline, every 5 and 15 min post-exercise in INT, and at corresponding time points in EX and SIT, day2: baseline, hourly	^a^; SBP: INT > SIT and EX during intermittent walking, lower 15 min post each walking, D2: INT and EX < SIT
Miyashita [10]	8 M (mean ± SE); age: 26.5 ± 1.5 y; BMI: 28.9 ± 1.4 kg/m^2^; SBP: 131 ± 4 DBP: 82 ± 5 mmHg	SIT: D1: 7 h sitting, day2: 6 h sitting; EX: day1: 30 min cycling @ 60% max HR after 30 min sitting, D2: 6 h sitting; 3. INT: day1: 10 × 3 min cycling @ 60% max HR, day2: 6 h sitting	Venepuncture on D2: 0 h, 2 h, 4 h, 6 h for TG, NEFA, 3OHB, insulin, plasma glucose	^a^ Postprandial: TAG (tAUC): EX and INT < SIT; INT and EX tending < SIT, main effect for 3OHB, exercise trials trending higher
Miyashita et al. [11]	10 M (mean ± SE); age: 24.4 ± 1.4 y, height: 176.8 ± 1.8 cm, weight: 71.2 ± 2.1 kg, BMI: 22.8 ± 0.6 kg/m^2^, WC: 78.0 ± 1.1 cm, bodyfat: 8.8% ± 0.7%, *V*O_2max_: 56.0 ± 4.1 ml · kg–1 · min–1 recreationally active	SIT: sitting 9 h; INT: 9 h sitting + 6 × 5 min running @ 70% *V*O_2max_, every 90 min, beginning 830am, last bout @ 4 pm	Cannula: 0 h, 1 h 30 min, 3 h, 4 h 30 min, 6 h, 7 h 30 min, 9 h for plasma TAG, glucose, insulin; serum CRP at 0 h, 9 h	^a^ CRP: ↔
Miyashita et al. [20]	Inactive, 15 W (mean ± SD); age: 68.8 ± 3.2 y, BMI: 24.0 ± 2.9 kg/m^2^ SBP: 135 ± 19 mm Hg DBP: 85 ± 10 mm Hg	1. SIT: 8 h sitting; 2. EX: 1 h sitting- > 30 min walking @ 3.7 ± 1.1 km/h, RPE: 12 ± 1 (0.33 ± 0.07 MJ/30 min)- > 6 h 30 min sitting 3. INT: 1 h sitting- > 20 × 1.5 min walking every 15 min @ 3.7 ± 1.1 km/h RPE: 11 ± 1	Venous: 0 h, 2 h, 4 h, 6 h, 8 h for TAG, NEFA, 3-OHB, insulin, glucose	^a^, NEFA: ↔; 3-OHB: INT > SIT, EX
Peddie et al. [62]	28 M 42 W (mean ± SD); age: (25.9 ± 5.3 y) BMI: 23.6 ± 4.0 kg/m^2^, questionnaire < 2.5 h/w (90 ± 42 min)PA; Healthy	1. SIT: 9 h sitting; 2. EX: 15 min sitting + 30 min treadmill walking @60% *V*O_2max_ (84.7BPM) + 8 h 15 min sitting 3. INT: 18 1min40 s (total 30 min) @ 45% *V*O_2max_ (85.6BPM) walking evenly spread over 9 h, same speed and incline as EX; 1st walk 15 min after 0 h	Cannula, 16 total: baseline, hourly, and 6 additional 30 and 45 min after meals for glucose, insulin, TAG	^a^
Pulsford et al. [21]	25 M (mean ± SD); age: 40.2 ± 12.2 y; inactive; BMI: 26.1 ± 4.1 kg/m^2^; BF: 26.6 ± 6%	SIT: 7 h sitting; STAND: sitting + 2 min standing every 20 min; WALK: sitting + 2 min walking (2mph) every 20 min	Cannula: − 1.5 h, OGTT @ 0 h, every 10 min for 30 min, then mixed meal @ 3 h, every 10 min for 30 min, every 30 min until 7 h for glucose, insulin, Matsuda index	^a^ Matsuda: WALK < SIT
Rodriguez-Hernandez et al. [82]	10 W (mean ± SE); age: 36 ± 5 y; BMI: 38.0 ± 5.66 kg/m^2^; bodyfat %: 49.57 ± 1.38%	SIT: 4 h sitting: WALK2 min: 2 min walking every 30 min, total 16 min, between 4 h sitting; WALK5 min: 5 min walking every 30 min, total 40 min, between 4 h sitting	4 h CGMS for glucose	^a^
Van Dijk et al. [83]	T2D patients (ADA criteria) 20 M (mean ± SD); age: 64 ± 1 y; BMI: 29.5 ± 0.9 kg/m^2^, PA unreported	SIT: 11 h? sitting; EX: sitting + breakfast + 45 min cycling @ 50% max workload capacity (EE: 350 kcal) + sitting; INT: sitting + 3 × 15 bouts of walking after each 3 meals (EE: 175 kcal)	Glucose CGMS; total 9 venous: 5 min before each meal, 90, 150 after each meal, last sample @ 1930 for glucose, insulin	Hyperglycaemia: EX < INT and SIT; ^a^
Vincent et al. [84]	6 M (mean ± SD); age: 27.0 ± 3.7 y; BMI: 24.8 ± 2.0 kg/m^2^	SIT: sleep restricted: D1, D2, D3: 700–200; INT: sleep restricted: D1, D2, D3: sitting 700–200, from 1000–1700, 3 min walking @ 3.2 km/h every 30 min, 51 min total walking	CGMS	
Wennberg et al. [22]	10 M 9 W (mean ± SD); age: 45–75 y, BMI: 31.5 ± 4.7 kg/m^2^; sitting time: 9.82 ± 2.19 h, MVPA ≤ 150 min/week	SIT: sitting 7 h; INT: sitting (402 min) + 3 min walking (3.2 km/h) every 30 min for 5 h, total 10	CGMS for glucose, cannula for insulin, BDNF, IL6, cortisol	^a^ ↔

^a^See Figs. 2, 4, 7

↔ no statistically significant difference between measures, **↑** increase; <  statistically significantly less than, e.g. if Glucose: Walk < Stand and Sit, this means AUC for glucose for the WALK condition was less than the STAND condition, and also less than the SIT condition, @ at, *D1* day 1, *D2* day2, *mean ± SD* mean ± standard deviation, *mean ± SE* mean ± standard error, *mean ± IQR* mean ± interquartile range, *RPE* rating of perceived exertion, *MET* metabolic equivalents, *CGMS* continuous glucose monitoring system, *T2D* type 2 diabetes, *OGTT* oral glucose tolerance test, *HFTT* high fat tolerance test, *FPG* fasting plasma glucose, *CVD* cardiovascular disease, *IFG* impaired fasting glucose, *GI* Glycaemic Index, CRP c reactive protein, LPL lipoprotein lipase, *NEFA* Non-esterified fatty acids, *HDL-C* high density lipoprotein cholesterol, LDL-C low density lipoprotein cholesterol, *TC* total cholesterol, *Apo A-I* apoliprotein A-I, *Apo B-48* apoliprotein B-48, *Apo B-100* apolipoprotein B-100, *SBP* systolic blood pressure, *DBP* dystolic blood pressure, *ABP* ambulatory blood pressure, *HR* heart rate, *3OHB* beta-hydroxy-butyrate, *BDNF* brain-derived neurotrophic factor, *IL6* interleukin 6, *HbA1c* haemoglobin A1c, *M* men, *W* women, *y* years old, *min* minute

#### Continuous/Prolonged vs PA Breaks

**Fig. 9:**
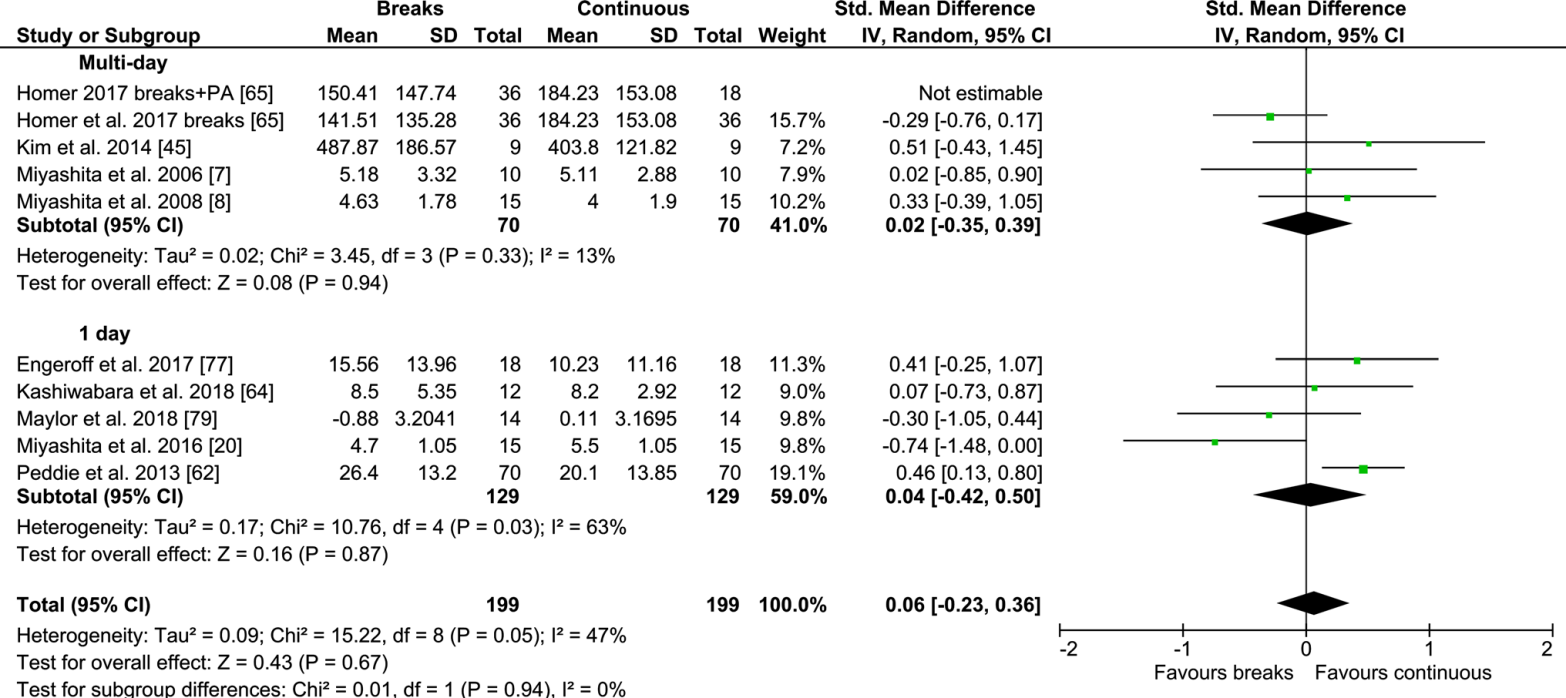
Forest plot for the effects of physical activity (PA) breaks vs continuous exercise on TAG measures, multi-day vs 1 day; D1: Day 1, d2: day 2

**Fig. 10:**
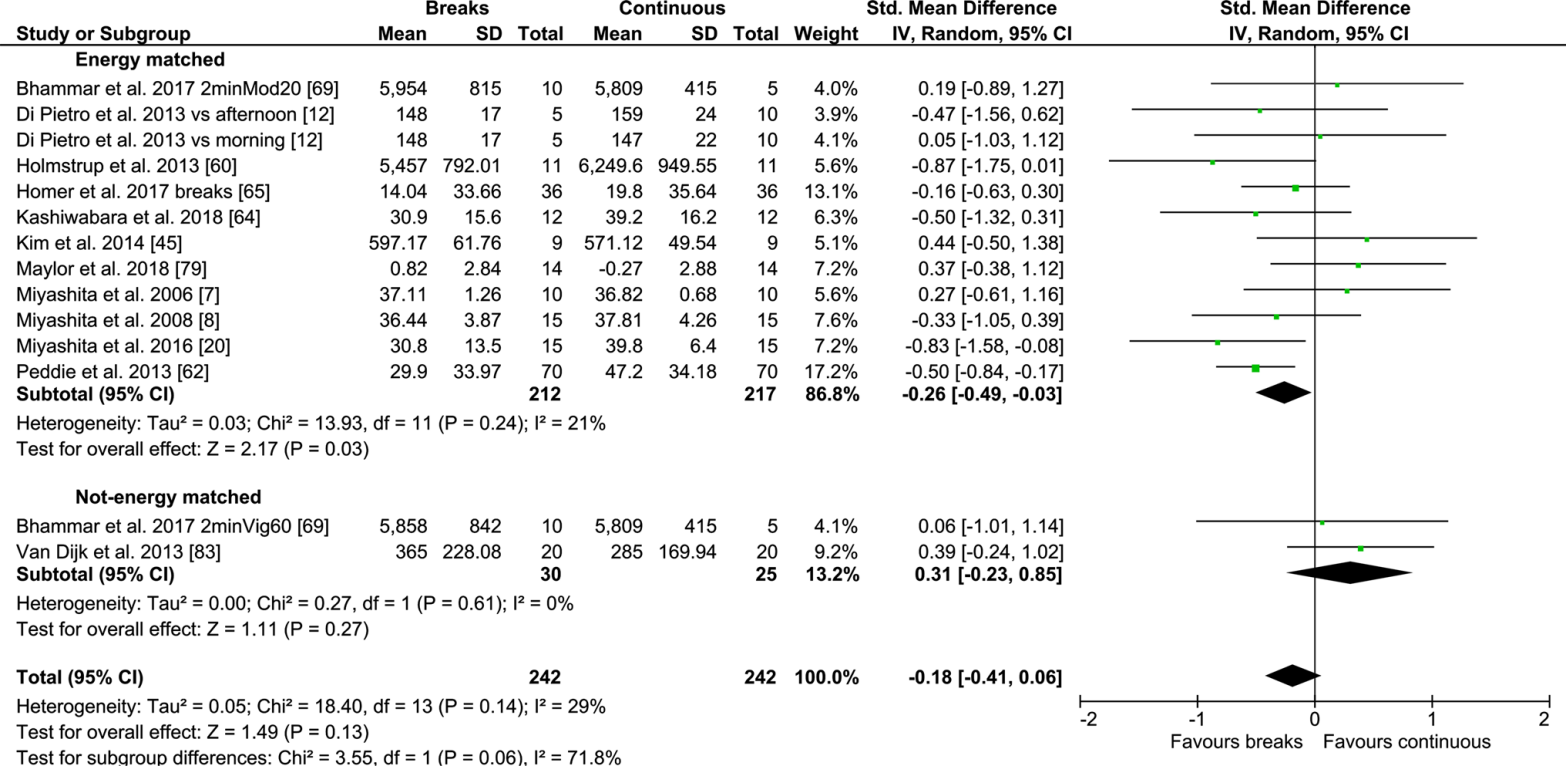
Forest plot for the effects of physical activity (PA) breaks vs continuous exercise on glucose measures

**Fig. 11:**
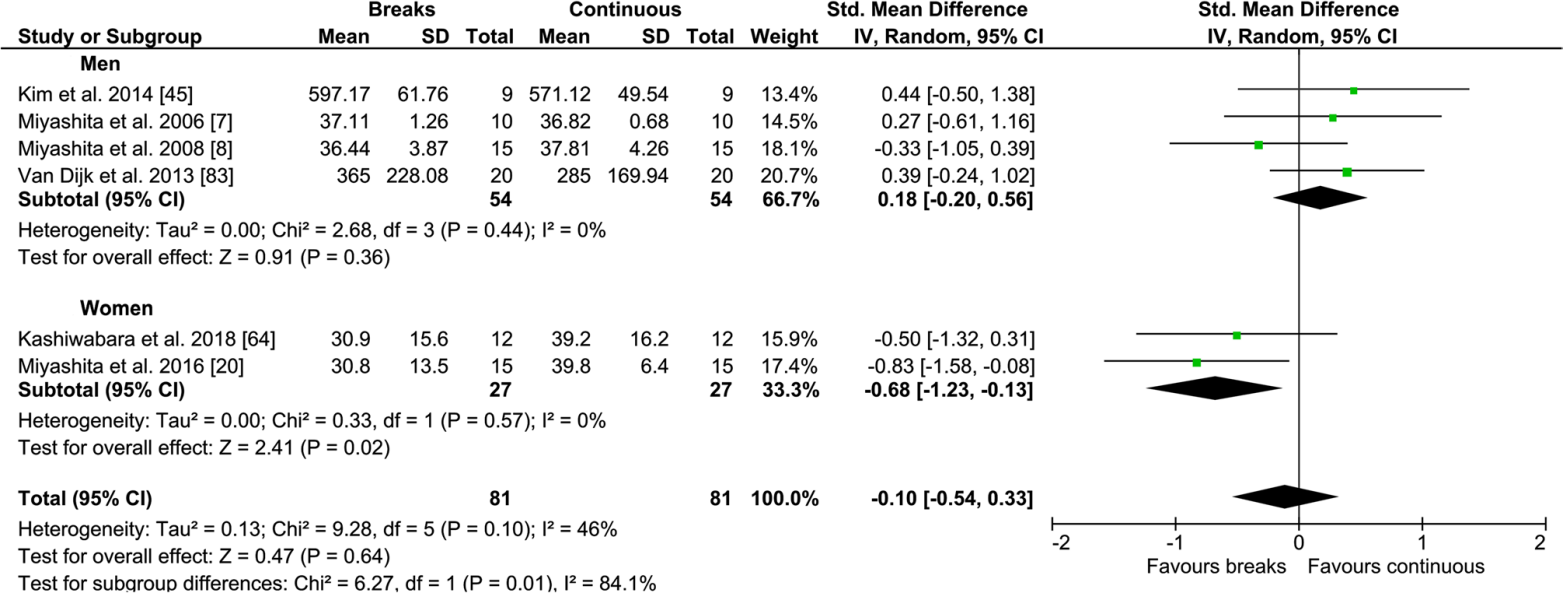
Forest plot for the effects of physical activity (PA) breaks on glucose measures, stratified by sex

**Fig. 12:**
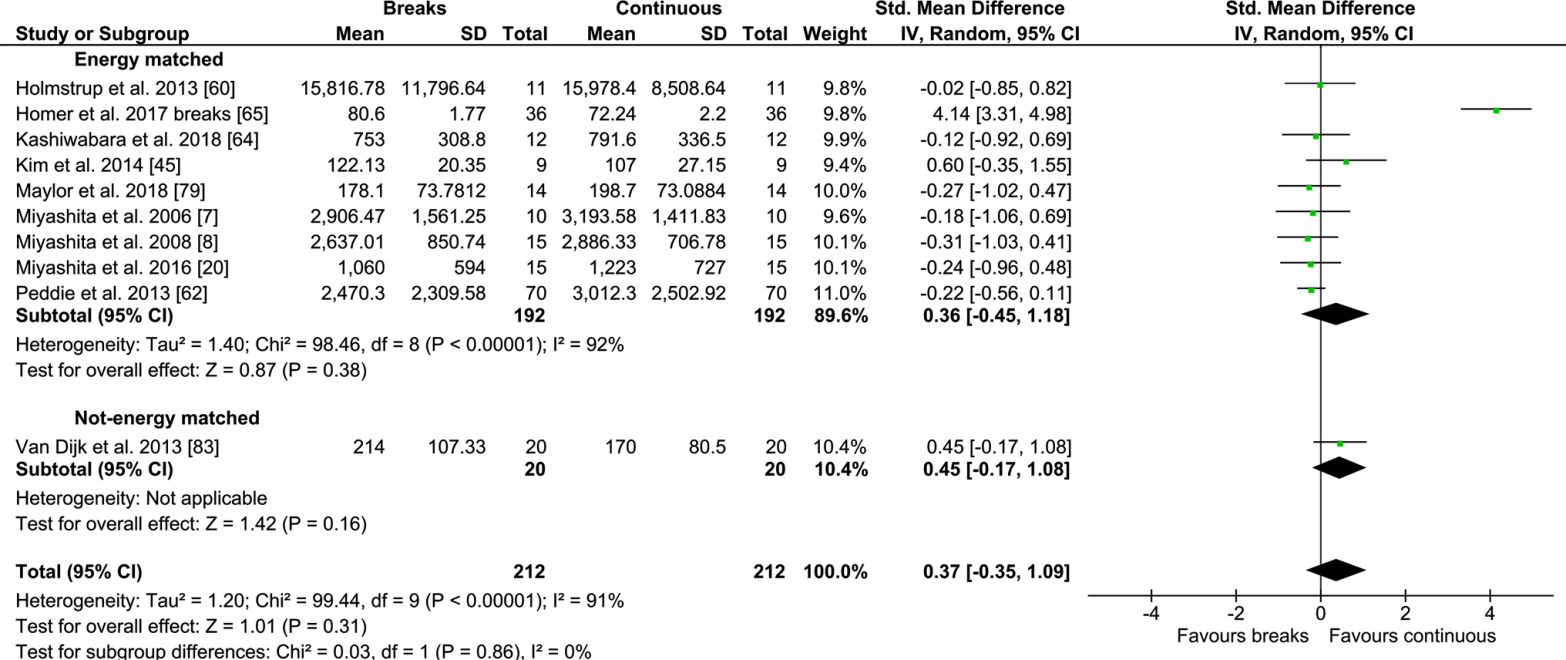
Forest plot for the effects of physical activity (PA) breaks vs continuous exercise on insulin measures

In total, 26 studies were reviewed (Table 2), of which 22 were meta-analysed. Participants ranged from those with type 2 diabetes [60] to those who were healthy and had relatively high levels of CRF [7, 8, 45]. The number of participants in studies ranged from 9 [45, 61] to 70 [62]. A total number of 232 participants were included in the meta-analysis for glucose outcomes, 212 for insulin outcomes and 199 for TAG outcomes. Participants were from 22.1 [8] to 70.5 years old [64]. Most studies utilised one day designs, but some utilised multi-day designs [7, 8, 45, 65]. Forest plots for TAG outcomes are presented in Fig. 9 and ‘Electronic Supplementary Material Appendix S2—Figs S3 and S4; for glucose outcomes in Figs. 10, 11 and Electronic Supplementary Material Appendix S2—Fig. S5’; and for insulin outcomes in Fig. 12 and Electronic Supplementary Material Appendix S2—Figs S6 and S7.

**Table 2:** Studies comparing PA breaks with 1 bout of continuous/prolonged exercise

Study	Participants	Protocol	Outcomes	Results
Bhammar et al. [69]	5 M 5 W (mean ± SD); age: M: 31 ± 5 y W: 32 ± 6 y; BMI: M: 30.1 ± 2.3 kg/m^2^, W: 30.5 ± 6.6 kg/m^2^; *V*O_2max_: M: 34.9 ± 4.0 ml/kg/min, W: 22.8 ± 2.7 ml/kg/min	SIT: 9 h sitting; 2 minMod20: 2min walking at 53 ± 5% HR max/3 miles/h every 20 min, total 42 min, 240 kcal. 2 minVig60: 2min walking at 79 ± 4% HR max every hour, total 16 min, 140 kcal; EX: 30 min walking at 71 ± 4% HR_max_/56% *V*O_2max_/3.3 miles/h, 230 kcal	CGMS. ABP, MAP	^a^, Systolic ABP, MAP: EX < SIT
Blankenship et al. [70]	2 M 8 W (mean ± SE); age: 51.9 ± 15.4 y; BMI: 31.6 ± 10.0 kg/m^2^, BF: 42.6 ± 3.3%	EX: 30 min brisk walking, ~ 300 kcal before lunch. FLB: isoenergetic with EX, bouts of sitting ≤ 20 min; FSB: bouts of sitting ≤ 20 min, same number of breaks as FLB but time walking standing reduced to minimise EE	CGMS, catheter for blood, after MMTT at end of day, @ 30, 60, 90, 120 min, for glucose, insulin	Post-prandial glucose and insulin AUC: ↔ between conditions; glycaemic variability: FLB < EX; nocturnal hyperglycaemia: FLB < EX and FSB
Di Pietro [12]	10 (mean ± SD); age: 69 ± 6 y; BMI: BMI 30 ± 5 kg/m^2^; impaired fasting glucose	INT: D1: inactive; D2: treadmill walking 3 × 15 min 3 METS postmeals; EXam: D1: inactive; D2: 45 min walking @ 3 METs @ 10.30 am; EXpm: D1: inactive; D2: 45 min walking @ 3 METs @ 430 pm	CGMS, glucose; insulin only on sitting days	Glucose: INT: d2 < d1; EXAM: d2 < d1; EXPM: ↔
Duvivier et al. [74]	2 M 16 W (mean ± SD); age: 21 ± 2 y; BMI: 22.6 ± 3.6 kg/m^2^; FPG: 4.61 ± 0.31 mmol/L	Over 4 days; SIT: 14 h sitting + 1 h walking + 1 h standing; EX: 13 h sitting + 1 h walking + 1 h standing + 1 h MVPA cycling; INT: 8 h sitting + 5 h walking + 3 h standing	Next day (day 5) fasting glucose, insulin, TAG, HDL-C, non-HDL-C, LDL-C, Apo-A, Apo-B; next day OGTT for IS	Glucose AUC/fasting: ↔; Insulin AUC: INT < SIT, INT < EX; fasting TAG: INT < SIT; fasting non-HDL-C: INT < SIT; Apo B: INT < SIT
Duvivier et al. [75]	13 M 6 W; (mean ± SD;)age: 63 ± 9 y, T2D (not on insulin), BMI: 30.5 ± 3.3 kg/m^2^, self-report MVPA: < 2.5 h/w, FPG: < 11 mmol/L	Over 4 days; SIT: 14 h sitting + 1 h walking + 1 h standing; EX: 13 h sitting + 1 h walking + 1 h standing + 1 h MVPA cycling (3 × 20 min bouts, 5 min rest between bouts); INT: 9 h sitting + 3 h walking + 4 h standing, after every 30 min sitting	Next day (day 5) 24 h CGM glucose; next day glucose, insulin fasting TAG, HDL-C, non-HDL-C, LDL-C, Apo-A, Apo-B	24 h iAUC GLUC: INT < SIT; Insulin: INT < SIT; HOMA2-IR: INT < SIT and EX; TG: INT and EX < SIT; C-peptide: INT < SIT; NEFA: SIT < INT and EX
Engeroff et al. [77]	Healthy, 18 W (mean ± SD); age: 25.6 ± 2.6 y, BMI: 21.5 ± 2.0 kg/m^2^*V*O_2max_: 41.3 ± 4.2 ml/kg/min; PA unreported	SIT: 4 h sitting; EX: 30 min cycling @ 70% VO2 max + 4 h sitting; INT: (40 min sitting + 6 min cycling @ 70% *V*O_2max_ + 40 min sitting	Venous TAG, TC, HDL-C, LDL-C, baseline, post 240 min	TAG: ↔ between conditions, overall time effect, **↑** for INT, SIT; TC: INT < EX; HDL-C: INT < EX, INT < SIT; LDL-C: ↔, **↑** for INT
Holmstrup et al. [60]	Obese, IFG, 8 M 3 W(mean ± SE); age: 25 ± 2.6 y, BMI: 34 kg/m^2^, Men *V*O_2max_: 32.6 ± 2.5 ml/kg/min, Women *V*O_2max_: 25.5 ± 1.8 ml/kg/min; light/moderate walking ≤ 5 × /w (questionnaire)	SIT: sitting: 12 h; EX: 1 h treadmill running @ 60–65% *V*O_2peak_, after baseline blood draw and 1st meal, sitting 11 h; INT 12 × 5 mins of treadmill running @ 60–65% *V*O_2peak_ every 1 h, 1st bout after baseline blood draw and 1st meal	Catheter, baseline, every 10 min over 12 h, for glucose, insulin, c-peptides	^a^; C-peptide: EX < SIT and INT during exercise, 2 h iAUC: EX and INT < SIT
Homer et al. [65]	11 M, 25 W (mean ± SD); age: 25 (19–34); BMI: 23.78 ± 4.01 kg/m^2^, *V*O_2max_: 36.19 ± mL/kg/min; Sedentary, < 150 min MVPA/w	SIT: D1: 7 h sitting, D2: 5 h sitting; EX: D1: 6 h 30 min sitting + 30 min walking @ 60% *V*O_2max_, D2: 5 h sitting; INT: D1 and D2: sitting + 2 min walking @ 60% *V*O_2max_ every 30 min; EX + INT: D1 and D2: sitting + 2 min walking @ 60% *V*O_2max_ every 30 min + 30 min walking @ 60% on D1	Cannula: D1: 0 h. D2: hourly + 30 min and 45 min post-meal, for TAG, glucose, insulin, NEFA	^a^; NEFA: ↔
Kashiwabara et al. [64]	12 W (mean ± SD); age: 70.5 ± 4.6 y; BMI: 25.3 ± 3.5 kg/m^2^; BP: 144 ± 19 mmHG; DBP: 85 ± 11 mmHG; inactive, < 150 min MVPA/w	SIT: 8 h sitting; INT: sitting + 1.5 min walking every 15 min @ 3.6 km/h, RPE: 11 @ 1 h, 1 h 15 min, 1 h 30 min, 1 h 45 min, 2 h 15 min, 2 h 30 min, 2 h 45 min, 4 h 15 min, 4 h 30 min, 4 h 45 min, 5 h, 5 h 15 min, 5 h 30 min, 5 h 45 min, 6 h 15 min, 6 h 30 min, 6 h 45 min, 7 h, 7 h 15 min, 7 h 30 min	Venepuncture: 0 h, 2 h, 4 h, 6 h, 8 h, for glucose, insulin, TAG, NEFA, APoB-48, ApoB-100, LPL	^a^; Apo B-48, Apo B-100, LPL: ↔
Kim et al. [45]	9 M (mean ± SD); age: 24.0 ± 4.0 y; *V*O_2max_: 51.6 ± 6.3 mL/kg/min; BMI < 30 kg/m^2^, recreationally active, healthy	SIT: D1 and D2: (7000–7500 steps/day, D3: 9 h sitting (< 2000 steps, 0900–1800), D4: HFTT; MOD: D1, D2, D4: same as SIT, D3: sitting +1 h running @ 65% *V*O_2max_ 3. INT: sitting + isoenergetic (with condition 2) intermittent walking, every hour, 9 sessions, 1st session 30 min, last session 60 min, 7 other sessions 17.8 ± 4.0 min) @ 25% *V*O_2max_ (total time: 214.5 min ± 28.0)	D4 fasting and postprandial FFA, TAG, glucose, insulin, indirect calorimetry for postprandial substrate oxidisation	^a^; FFA: MOD > INT and SIT
Maylor et al. [79]	7 M and7 W (mean ± SD); age: 29 ± 9 y, BMI: 26.1 ± 5.8 kg/m^2^, *V*O_2max_: 38.6 ± 4.2 mL/kg/min; Sedentary, inactive,	SIT: 8H sitting; EX: 30 min sitting + 30 min treadmill running @ 60% VO2 reserve + 7 h sitting; INT: 30 min sitting + 2 min 32 s running @ 85% VO2 reserve every 60 min, 8 bouts	Cannula: 0 h. hourly intervals, for TAG, glucose, insulin, HDL-C	^a^; HDL-C: INT < SIT
Miyashita et al. [7]	10 M (mean ± SE); age: 25.0 ± 1.3 y, BMI: 25.4 ± 1.2 kg/m^2^, WC: 87.2 ± 3.5 cm, BF: 9.4 ± 0.7% *V*O_2max_: 56.3 ± 1.8 mL/kg/min; Healthy, recreationally active	1.SIT: D1: 7 h sitting, D2: 7 h sitting; 2. EX: D1: 6 h 30 min sitting + 30 min running @ 71.1 ± 2.3% *V*O_2max_; D2: 7 h sitting; 3: INT: D1: 10 × 3 min running @ 69.6 ± 1.0% *V*O_2max_ between every 30 min of sitting over 7 h, D2: 7 h sitting	D2 cannula: 0 h, hourly intervals, and @ 0.5, 0.75, 3.5, 3.75 h fasting and post-prandial for TAG, glucose, insulin, NEFA, 3-OHB	^a^; NEFA, 3-OHB: ↔
Miyashita et al. [8]	15 M; (mean ± SE): age: 23.4 ± 0.8 y, *V*O_2max_: 56.3 ± 2.1 mL/kg/min, BMI: 23.4 ± 0.6 kg/m^2^, WC: 80.8 ± 2.1 cm, BF: 11.2 ± 0.9%, SBP: 114 ± 2 mm Hg, DBP: 68 ± 2 mm Hg; non-smoking, BP < 140/90 mmHg,	1. SIT: D1: 7 h sitting, D2: 7 h sitting; 2: day1: EX: 30 min walking @ 6.8 km/h ± 0.1 (42.4 ± 1.8% *V*O_2max_) after 6 h 30 min sitting, day2: 7 h sitting; 3: INT: D1: 10 × 3 min walking 6.8 km/h ± 0.1 (41.4 ± 1.8% *V*O_2max_) between every 30 min of sitting over 7 h, D2: 7 h sitting	Next day TAG, glucose, insulin; BP: day1: baseline, every 5 and 15 min post-exercise in INT, and at corresponding time points in EX and SIT, day2: baseline, hourly	^a^; SBP: INT > SIT and EX during intermittent walking, lower 15 min post each walking, D2: INT and EX < SIT
Miyashita et al. [20]	Inactive, 15 W (mean ± SD); age: 68.8 ± 3.2 y, BMI: 24.0 ± 2.9 kg/m^2^ SBP: 135 ± 19 mm Hg DBP: 85 ± 10 mm Hg	1. SIT: 8 h sitting; 2. EX: 1 h sitting- > 30 min walking @ 3.7 ± 1.1 km/h RPE RPE 12 ± 1 (0.33 ± 0.07 MJ/30 min)- > 6 h 30 min sitting 3. INT: 1 h sitting- > 20 × 1.5 min walking every 15 min @ 3.7 ± 1.1 km/h RPE: 11 ± 1	Venous: 0 h, 2 h, 4 h, 6 h, 8 h for TAG, NEFA, 3-OHB, insulin, glucose	^a^, NEFA: ↔; 3-OHB: INT > SIT, EX
Peddie et al. [62]	28 M 42 W (mean ± SD); age: (25.9 ± 5.3 y) BMI: 23.6 ± 4.0 kg/m^2^, questionnaire < 2.5 h/w (90 ± 42 min)PA; Healthy	1. SIT: 9 h sitting; 2. EX: 15 min sitting + 30 min treadmill walking @60% *V*O_2max_ (84.7BPM) + 8 h 15 min sitting 3. INT: 18 1min40 s (total 30 min) @ 45% *V*O_2max_ (85.6BPM) walking evenly spread over 9 h, same speed and incline as EX; 1st walk 15 min after 0 h	Cannula, 16 total: baseline, hourly, and 6 additional 30 and 45 min after meals for glucose, insulin, TAG	^a^
Van Dijk et al. [83]	T2D patients (ADA criteria) 20 M (mean ± SD); age: 64 ± 1 y; BMI: 29.5 ± 0.9 kg/m^2^, PA unreported	SIT: 11 h? sitting; EX: sitting + breakfast + 45 min cycling @ 50% max workload capacity (EE: 350 kcal) + sitting; INT: sitting + 3 × 15 bouts of walking after each 3 meals (EE: 175 kcal)	Glucose CGMS; total 9 venous: 5 min before each meal, 90, 150 after each meal, last sample @ 1930 for glucose, insulin	Hyperglycaemia: EX < INT and SIT;^a^

^a^See Figs. 9, 10, 12

*↔* no statistically significant difference between measures, **↑** increase; <  statistically significantly less than, e.g. if Glucose: Walk < Stand and Sit, this means AUC for glucose for the WALK condition was less than the STAND condition, and also less than the SIT condition, @ at, *D1* day 1, *D2* day2, *mean ± SD* mean ± standard deviation, *mean ± SE* mean ± standard error, *mean ± IQR* mean ± interquartile range, *RPE* rating of perceived exertion, *MET* metabolic equivalents, *CGMS* continuous glucose monitoring system, *T2D* type 2 diabetes, *OGTT* oral glucose tolerance test, *HFTT* high fat tolerance test, *FPG* fasting plasma glucose, *CVD* cardiovascular disease, *IFG* impaired fasting glucose, *GI* Glycaemic Index, *CRP* c reactive protein, *LPL* lipoprotein lipase, *NEFA* non-esterified fatty acids, *HDL-C* high density lipoprotein cholesterol, *LDL-C* low density lipoprotein cholesterol, *TC* total cholesterol, *Apo A-I* apoliprotein A-I, *Apo B-48* apoliprotein B-48, *Apo B-100* apolipoprotein B-100, *SBP* systolic blood pressure, *DBP* dystolic blood pressure, *ABP* ambulatory blood pressure, *HR* heart rate, *3OHB* beta-hydroxy-butyrate, *BDNF* brain-derived neurotrophic factor, *IL6* interleukin 6, *HbA1c* haemoglobin A1c, *M* men, *W* women, *y* years old, *min* minute

Duvivier et al. [74–76], and Blankenship [70] were not included in the meta-analysis as the PA breaks protocol were not clearly stated, and free-living designs were used; however, they were included in the narrative summary (Table 2). All but one [77] study had participants randomised into crossover trial conditions.

### Primary Outcomes

#### Physical Activity Breaks vs Sitting

Overall, there was a small but statistically significant effect for TAG outcomes, an SMD of − 0.26 (95% CI − 0.44, − 0.09, *p *= 0.002) (Fig. 2). There were statistically significant moderate effects for PA breaks on glucose [SMD − 0.54 (95% CI − 0.70, − 0.37, *p* = 0.00001) (Fig. 4)] and insulin [SMD 0.56 (95% CI − 0.74, − 0.38, *p* = 0.00001) (Fig. 7)].

#### Meta-regression

**Fig. 13:**
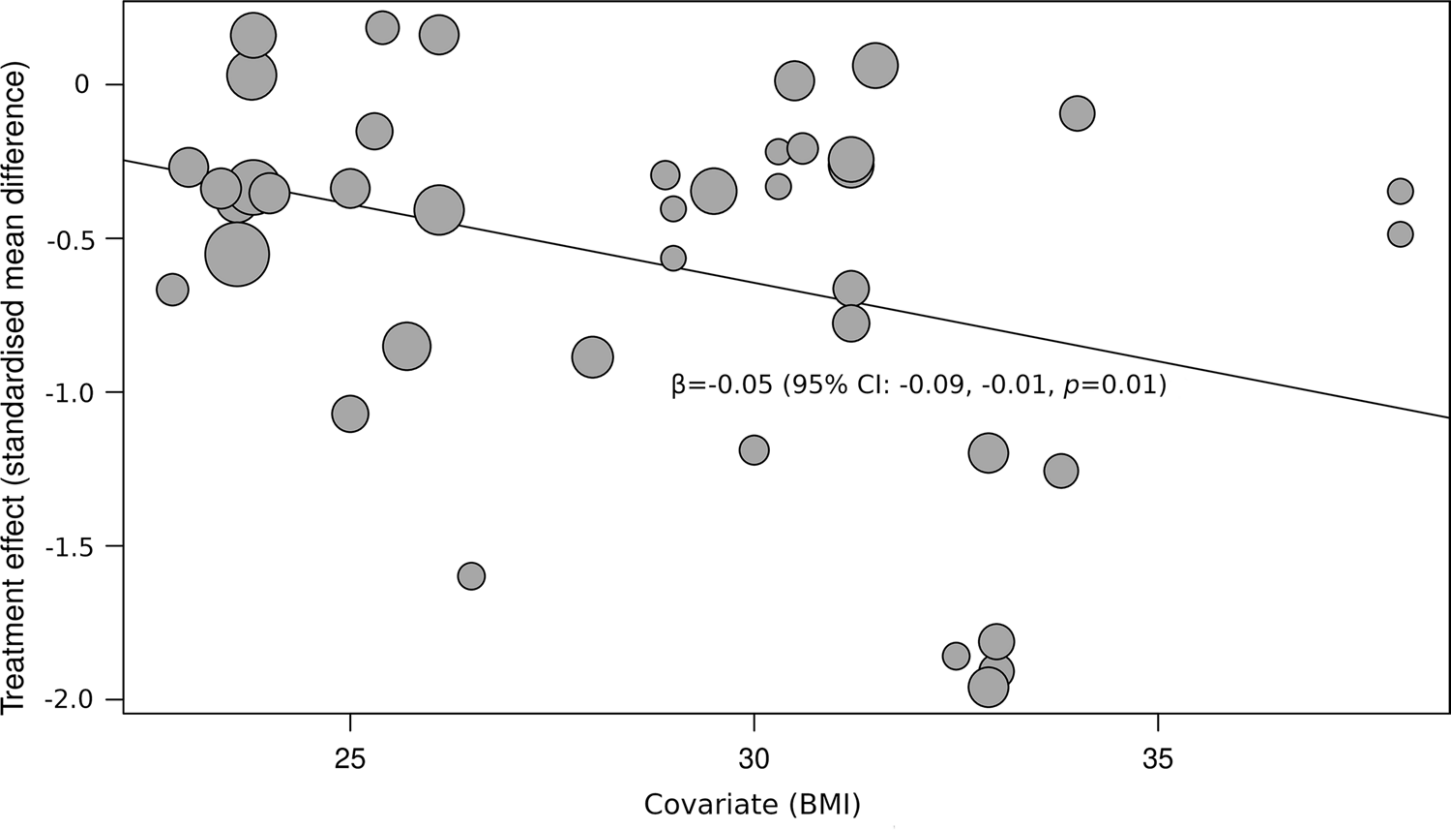
Bubble plot illustrating the association between BMI and SMD when PA breaks were compared with sitting on blood glucose measures. A bubble represents a study. A negative value for SMD means that PA breaks resulted in lower blood glucose values, a positive SMD indicates that sitting resulted in lower glucose values

**Fig. 14:**
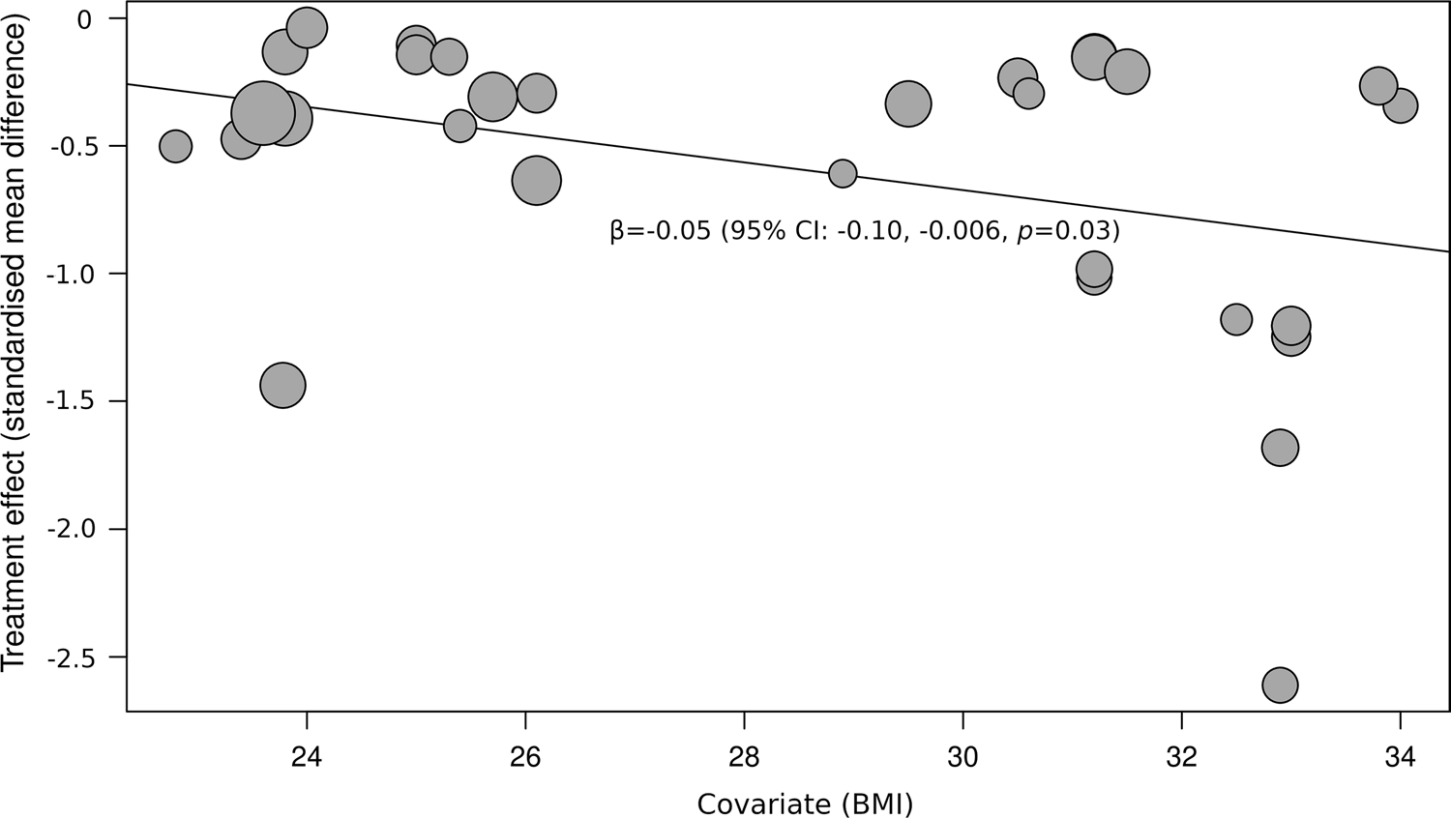
Bubble plot illustrating the association between BMI and SMD when PA breaks were compared with sitting on blood glucose measures. A bubble represents a study. A negative value for SMD means that PA breaks resulted in lower blood glucose values, a positive SMD indicates that sitting resulted in lower glucose values

BMI was statistically significantly associated with glucose (*β* = − 0.05, 95% CI − 0. CI − 0.09, − 0.01, *p *= 0.01) (Fig. 13) and insulin (*β* = − 0.05, 95% CI − 0.10, − 0.006, *p *= 0.03) (Fig. 14) responses to PA breaks compared with sitting, suggesting that the observed effects were larger in more obese participants. TAG (*β* = 0.02, 95% CI − 0.02, 0.06, *p *= 0.37) responses were not associated with BMI. Bailey et al. [68] and Kim et al. [45] were not included in the meta-regression, as BMI was not reported.

#### Publication Bias

**Fig. 15:**
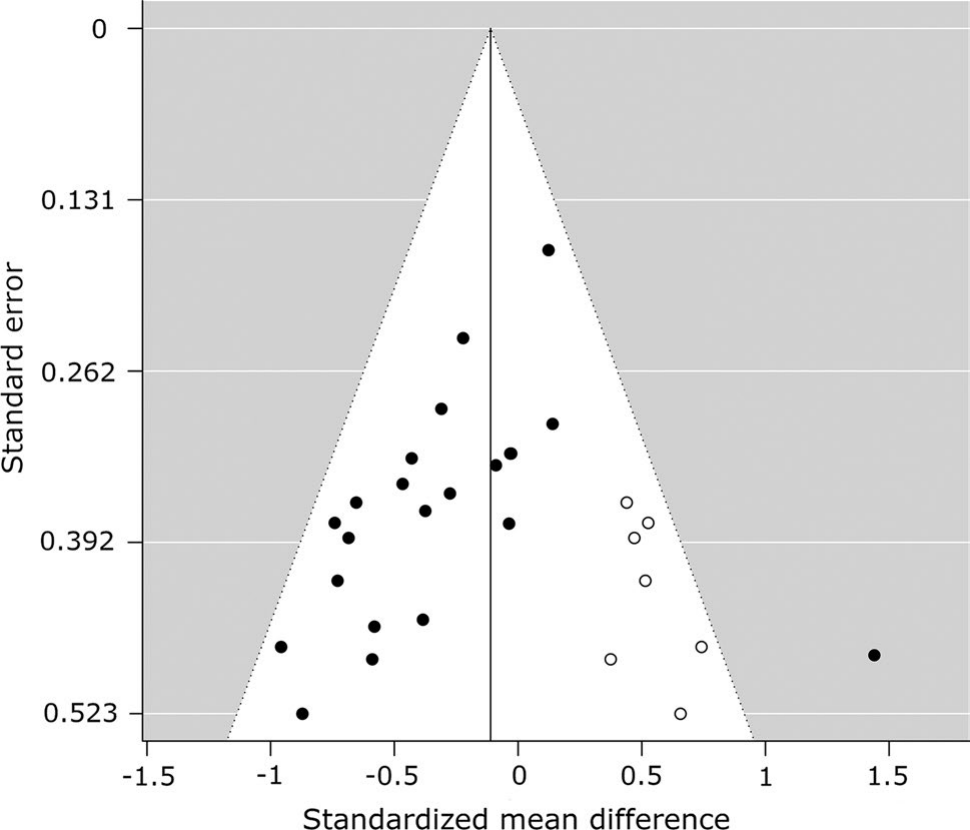
Funnel plot for triacylglycerol measures, random-effects model: physical activity breaks versus sitting. A filled circle represents a study; an empty circle, if present, represents a “missing” study by the trim and fill method

**Fig. 16:**
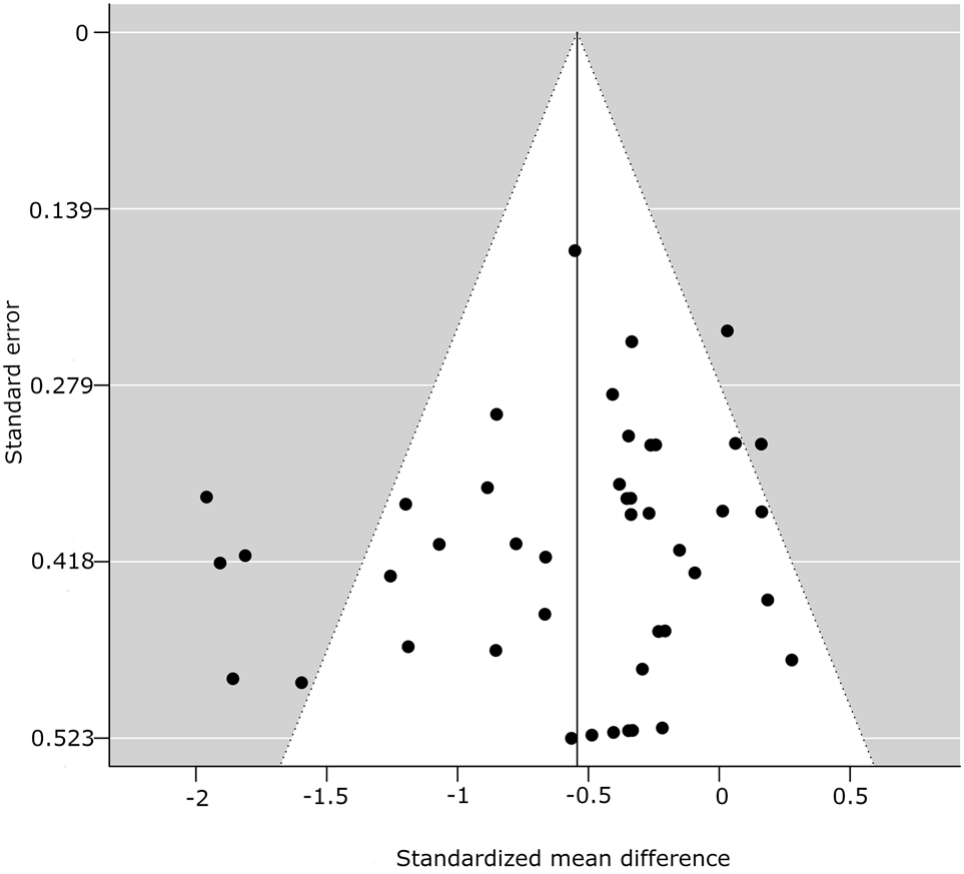
Funnel plot for glucose measures, random-effects model: physical activity breaks versus sitting. A filled circle represents a study; an empty circle, if present, represents a “missing” study by the trim and fill method

**Fig. 17:**
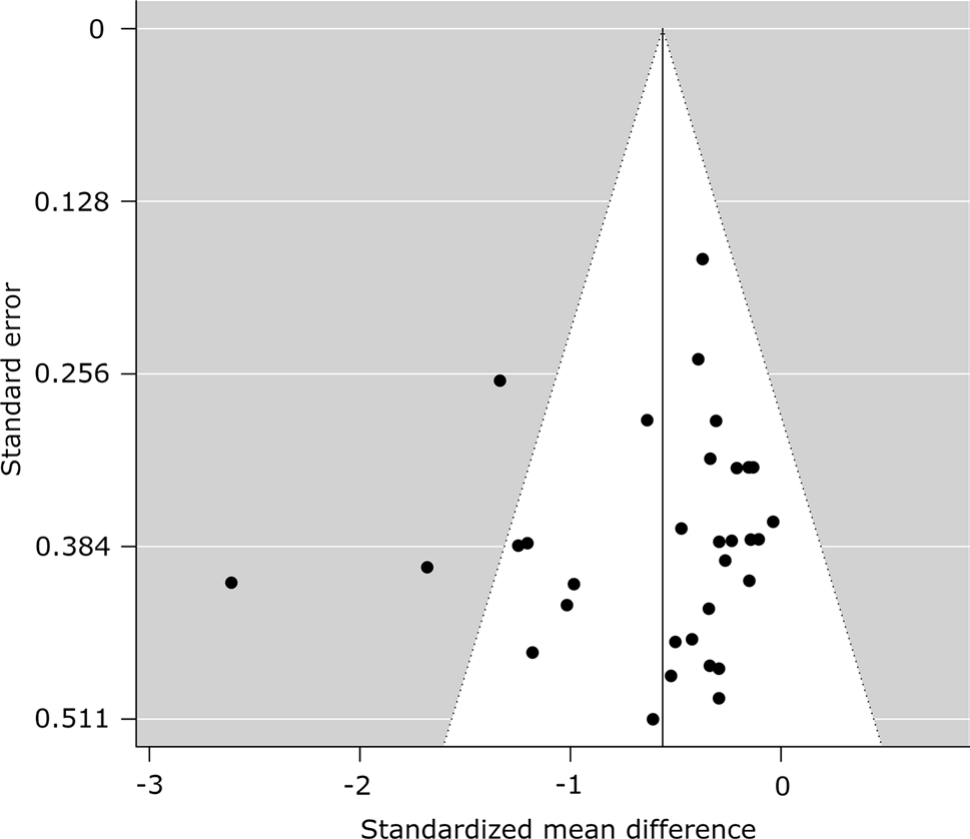
Funnel plot for insulin measures, random-effects model: physical activity breaks versus sitting. A filled circle represents a study; an empty circle, if present, represents a “missing” study by the trim and fill method

There was an asymmetrical funnel plot for TAG (Fig. 15) outcomes when PA breaks were compared to sitting, but not for glucose (Fig. 16) or insulin (Fig. 17), suggesting the possible existence of publication bias for TAG outcomes (Table 3).

**Table 3:** Statistical tests for publication bias for the meta-analyses of glucose, insulin, and TAG levels: physical activity breaks vs sitting, physical activity breaks vs continuous exercise

Metabolic variable	Rosenthal’s fail-safe *N*	Begg and Mazumdar (*p* value)	Egger (*t* value, *p* value)	SMD, assuming severe 2-tailed selection bias
Glucose, breaks vs sitting	1358	0.09	− 1.25, 0.22	− 0.41
Insulin, breaks vs sitting	907	0.03	− 0.80, 0.43	− 0.43
TAG, breaks vs sitting	87	0.005	− 2.09, 0.05	− 0.20

*SMD* standardised mean difference

### Secondary Outcomes

#### Continuous/Prolonged Exercise vs PA Breaks

There were no statistically significant differences for TAG outcomes, with an SMD of 0.08 (95% CI − 0.22, 0.37, *p *= 0.62) (Fig. 9), or insulin (Fig. 12), with an SMD of 0.35 (95% CI − 0.37, 1.07, *p *= 0.35), but there was a statistically significant small to moderate effect for glucose with an SMD of − 0.26 (95% CI − 0.50, − 0.02, *p *= 0.03) (Fig. 10), as a result of intermittent PA breaks compared to one bout of continuous exercise in the context of prolonged sitting. Only two studies [77, 79] compared lipoprotein responses to PA breaks and continuous exercise (Table 2), with PA breaks decreasing high-density lipoprotein (HDL) cholesterol in comparison to sitting [77, 79] and continuous exercise [77].

#### Meta-regression and Publication Bias

There was no association between BMI and glucose SMD (*β* = 0.008, 95% CI − 0.06, 0.08, *p* = 0.81) for PA breaks versus one bout of continuous exercise. No meta-regression was performed for insulin and TAG measures due to the small number of studies [85, 86].

There was a possible publication bias for insulin measures (Table 4).

**Table 4:** Statistical tests for publication bias for the meta-analyses of glucose, insulin, and TAG levels, physical activity breaks vs sitting

Metabolic variable	Rosenthal’s fail-safe *N*	Begg and Mazumdar (*p* value)	Egger (*t* value, *p* value)	SMD, assuming severe 2-tailed selection bias
Glucose, breaks vs continuous	12	0.27	1.61, 0.13	− 0.17
Insulin, breaks vs continuous	0	0.00009	1.06, 0.32	0.14
TAG, breaks vs continuous	0	1.0000	− 1.35, 0.21	0.02

*SMD* standardized mean difference

### Risk of Bias

Other than a few studies [15, 17, 21, 46, 47] most did not utilise or report any form of blinding. All studies included in the meta-analysis, except one [77], were randomised, but only a few reported the randomisation methods clearly [17, 19, 21, 22, 46, 47, 62]. Additionally, with the exception of a few studies [15, 17, 19, 46, 47, 62], most did not report how any possible missing data were handled. Notably, studies with the most rigorous design or reporting [15, 17, 46, 47] appeared to report larger effects, for example, on glucose and insulin (Figs. 4, 7) (Table 5).

**Table 5:** Risk of bias summary for included studies

Study	Random sequence generation (selection bias)	Allocation concealment (selection bias)	Blinding of participants and personnel (performance bias)	Blinding of outcome assessment (detection bias)	Incomplete outcome data (attrition bias)	Selective reporting (reporting bias)	Other bias
Bailey et al. [68]	?	?	N	N	Y	Y	Y
Bailey et al. [13]	?	?	N	N	Y	Y	Y
Bailey et al. [63]	?	?	N	N	Y	Y	Y
Bailey et al. [63]	?	?	N	N	Y	Y	Y
Bhammar et al. [69]	?	?	N	N	Y	Y	Y
Blakenship et al. [70]	?	?	N	N	Y	Y	Y
Brocklebank et al. [71]	Y	Y	N	N	Y	Y	Y
Champion et al. [72]	?	?	N	N	Y	Y	Y
Chen et al. [73]	?	?	N	N	Y	Y	Y
Crespo et al. [14]	?	?	N	N	Y	Y	Y
Dempsey et al. [15, 47]	Y	Y	?	Y	Y	Y	Y
Di Pietro et al. [12]	?	?	N	N	Y	Y	Y
Dunstan et al. [46]	Y	Y	?	Y	Y	Y	Y
Duvivier et al. [74]	?	?	N	N	Y	Y	Y
Duvivier et al. [75]	Y	Y	N	Y	Y	Y	Y
Duvivier et al. [76]	Y	Y	N	Y	Y	Y	Y
Engeroff et al. [77]	N	N	N	N	Y	N	Y
Hansen et al. [16]	?	?	N	N	Y	Y	Y
Hawari et al. [78]	?	?	N	N	Y	Y	Y
Henson et al. [17]	?	?	?	Y	Y	Y	Y
Holmstrup et al. [60]	?	?	N	N	Y	N	?
Homer et al. [65]	?	?	N	N	Y	Y	Y
Honda et al. [18]	?	?	N	N	Y	Y	Y
Kashiwabara et al. [64]	?	?	N	N	Y	Y	Y
Kerr et al. [61]	?	?	N	N	Y	Y	Y
Kim et al.	?	?	N	N	Y	N	Y
Larsen et al. [19]	Y	Y	?	N	Y	N	Y
Maylor et al. [79]	?	?	N	N	Y	Y	Y
McCarthy et al. [80]	?	?	N	N	Y	Y	Y
McCarthy et al. [81]	?	?	N	N	Y	Y	Y
Miyashita et al. [7]	?	?	N	N	Y	N	Y
Miyashita et al. [9]	?	?	N	N	Y	Y	Y
Miyashita et al. [8]	?	?	N	N	Y	N	Y
Miyashita et al. [10]	?	?	N	N	Y	N	Y
Miyashita et al. [11]	?	?	N	N	?	?	Y
Miyashita et al. [20]	?	?	N	N	Y	Y	Y
Peddie et al. [62]	Y	Y	N	N	Y	N	Y
Pulsford et al. [21]	Y	Y	?	N	Y	N	Y
Rodriguez-Hernandez et al. [82]	?	?	N	N	Y	N	Y
Van Dijk et al. [83]	?	?	N	N	Y	N	Y
Vincent et al. [87]	?	?	N	N	Y	N	Y
Wennberg et al. [22]	Y	Y	N	N	Y	N	Y

*Y* not at risk of bias for this condition, *N* at risk of bias for this condition, *?* risk of bias for this condition is unknown based on the reported methodology

## Discussion

### Main Findings

#### Physical Activity Breaks vs Sitting

Overall, there were statistically significant differences between PA breaks (INT) compared to sitting (SIT) on measures of glucose, insulin and TAG. The effect for TAG was small, SMD of − 0.27 − 0.26 (95% CI − 0.44, − 0.09, *p *= 0.002), whereas the effects for glucose, SMD of − 0.54 (95% CI − 0.70, − 0.37, *p *= 0.00001), and insulin, SMD of − 0.56 (95% CI − 0.74, − 0.38, *p *= 0.00001) were moderate. The observed effects on glucose (*β* = − 0.05, 95% CI − 0.09, − 0.01, *p* = 0.01), and insulin (*β* = − 0.05, 95% CI − 0.10, − 0.006, *p* =− 0. 0.03) responses were more pronounced in participants with larger BMIs. A negative β coefficient indicates that as BMI increases, the SMD between PA breaks compared to sitting is negative, with a negative SMD indicating an effect in favour of breaks. The small effect of breaks on TAG could be due to the delayed effects of exercise on lipids [39, 40]. Whereas studies using single day designs reported no statistically significant effects, those with two or multi-day designs did (Fig. 2). Heterogeneity in some of the meta-analyses might be explained by differences in study population and design, as discussed in Sect. 4.3.

#### Continuous/Prolonged Exercise vs Intermittent

Overall, the meta-analysis found no statistically significant differences between prolonged/continuous exercise compared to PA breaks in sitting on postprandial insulin and TAG. Notably, PA breaks had a greater effect on glycaemia in studies that were energy matched (Fig. 10), with a small to moderate effect: − 0.26 (95% CI − 0.50, − 0.02, *p *= 0.03).

### Implications

Several short-term experimental studies have shown that PA breaks attenuate post-prandial increases in glucose (Fig. 4) and insulin (Fig. 7) on the same day, compared to no-exercise sitting. Additionally, these effects persisted overnight [14, 15]. The sustained effects of PA breaks warrant further research, especially with the increasing use and availability of CGMS. The effects of breaks on TAG were weaker, but PA breaks still appear to attenuate TAG somewhat (Fig. 2). Physically inactive or sedentary participants or those with IFG or T2D experienced greater benefits in glycaemic attenuation (Fig. 5), as did those with higher BMI, as revealed by meta-regression (Sect. 3.3.2) (Fig. 13).

To place these results in the wider context of the effects of exercise on markers of metabolic health, in a meta-analysis of non-laboratory based randomised controlled trials of PA interventions lasting from 2 to 6 months in people with type 2 diabetes aged 35–71 years, walking, yoga, tai chi and qigong had a cumulative SMD of − 0.60 (95% CI − 0.83, − 0.37) compared with no exercise on glycaemic control, as indicated by glycated haemoglobin (HbA1c) [88]. High intensity interval training (HIIT) interventions lasting more than 2 weeks, compared to no exercise, reduced insulin resistance by an SMD of − 0.49 (95% CI − 0.87, − 0.12) in all groups, by − 0.38 (95% CI − 1.39, 0.63) in overweight/obese, and by − 0.62 (95% CI − 1.10, − 0.14) in people with type 2 diabetes [89]. Similarly, short term HIIT, lasting less than 12 weeks, reduced fasting glucose by an SMD of − 0.35 (95% CI − 0.62, 0.0.09) in overweight or obese people [90]. Additionally, in people with non-alcoholic fatty liver disease, exercise interventions, whether aerobic, resistance, or combined, lasting more than 1 month, reduced the glucose parameters HbA1c and homeostatic model of assessment of insulin resistance (HOMA-IR) by SMDs of − 0.76 (95% CI − 0.78, − 0.42) and − 0.50 (95% CI − 0.85, − 0.15), respectively [91], compared to normal care. Similarly, exercise reduced postprandial total TAG by Cohen’s *d* of − 0.60 (95% CI − 0.69, − 0.50), and iAUC TAG by − 0.59 (95% CI − 0.76, − 0.42) in all participants [92]. Since all but one of these meta-analyses [92] were not laboratory based and evaluated acute or longer-term protocols and adherence to the exercise protocols was less easy to confirm, they should be compared with our findings only generally and cautiously. Similarly, a previous meta-analysis [6] of 5 studies [46, 60, 62, 68, 83] reported PA breaks resulted in lower glucose measures than sitting. The effect sizes reported in our current meta-analysis, whether for measures of glucose, insulin, or TAG, can be seen to be generally similar to the effect sizes of diverse exercise modalities in various populations reported in the literature.

Recently, a meta-analysis [93] reported that activity breaks compared to sitting lowered post-prandial glucose by Cohen’s d of − 0.36 (95% CI − 0.50, − 0.21), and postprandial insulin by Cohen’s d of − 0.37 (95% CI − 0.53, − 0.20). The mean postprandial TAG response with breaks was reduced by 0.06 (95% CI − 0.15, 0.26) compared with sitting. The findings of the meta-analysis by Saunders et al. [93] for glucose and insulin outcomes were broadly similar to our findings, but with smaller effect sizes. However, we found that PA breaks lowered post-prandial TAG outcomes, in contrast to Saunders et al. [93]. The differences in results could be explained by differences in inclusion criteria. Saunders et al. included adolescents and teenagers [94–98] in their meta-analysis [93], whereas we did not. Furthermore, whereas we included studies with people with type 2 diabetes in our meta-analysis, Saunders et al. [93] did not. It is possible that we found that PA breaks compared to sitting had greater benefits on glucose, insulin and TAG outcomes than Saunders et al. [93] because participants in their meta-analysis were healthier and younger. This is supported generally by our meta-regression and subgroup analyses, which suggested that people with higher BMI, lower cardiovascular fitness, impaired fasting glucose or type 2 diabetes, experienced greater reductions in post-prandial glucose and insulin, compared to those with lower BMI or who were healthier. However, Saunders et al. [93] reported that neither glucose nor insulin outcomes were associated with BMI. This discrepancy between their findings and ours might again be due to the younger and healthier participants in their analyses, as transport, uptake and metabolism of glucose might be greater in the insulin sensitive compared to the insulin resistant [44]. Additionally, whereas Saunders et al. only included studies involving bouts of light to moderate activity, we did not limit studies based on exercise intensity. Moreover, we also performed meta-analyses of PA breaks in comparison to one continuous bout of exercise, reporting a small effect in favour of PA breaks on post-prandial glucose outcomes.

These post-prandial effects of PA breaks on measures of glucose, insulin and TAG could be relevant to the prevention of type 2 diabetes and atherosclerosis. The post-prandial [99] state is the more common metabolic state during non-sleeping hours for many people in modern society, who consume three large meals a day in addition to snacks and drinks [100]. Post-prandial and nocturnal hyperglycaemic excursions might be an early and undetected aspect of an insulin-resistant state [101]. Hyperglycaemic spikes are more strongly associated with, and might be more predictive of cardiovascular complications, risk and all-cause mortality than fasting plasma glucose or HbA1c levels [102] and should be targeted [103] since HbA1c is an integrative measure of blood glucose and post-prandial hyperglycaemia occurs even when HbA1c control is adequate [104]. Notably, post-load glucose-predicted cardiovascular mortality and diabetes, whereas neither fasting glucose nor HbA1c did [105]. Additionally, elevated 30 min post-load glycaemia is associated with increased risk of type 2 diabetes and all-cause mortality, independent of both fasting and 2 h post-load glucose [106]. Similarly, post-load insulin levels during a glucose tolerance test predict the development of type 2 diabetes [107], as insulin release is pulsatile, resulting in oscillating ultradian periodicity [108, 109]. Similarly, post-prandial excursions in TAG also increase CVD risk [110–114], via atherogenesis [115]. Therefore, the moderate decreases in post-prandial glucose and insulin, and the small decrease in post-prandial TAG, as a result of PA breaks in sitting, if confirmed in longer-term studies, may have implications for the prevention of metabolic disease, at least in comparison with only sitting.

This meta-analysis suggests any differences in metabolic effects between regular PA breaks and one continuous bout of exercise are non-existent for TAG (Fig. 9) and insulin (Fig. 12), or statistically significant but small for glucose (Fig. 10). In a previous meta-analysis [6], MPA breaks were more effective than a single prolonged bout of MPA at regulating glycaemia, even when the study in which the continuous bout resulted in double the amount of energy expended compared to the intermittent bout was included [6]. However, only three studies [60, 62, 83], two of which were energy matched [60, 62], were meta-analysed [6]. In our current meta-analysis, when EE was matched, there was a small and statistically significant effect in favour of regular PA breaks on post-prandial glycaemia (Fig. 10). In the largest meta-analysis of observational studies to date, the increased risk of all-cause and CVD mortality associated with high sitting time, specifically sitting for more than 8 h daily, was entirely eliminated by approximately 60-75 min daily, and reduced by approximately 30 min daily, of self-reported PA [116]. PA breaks in the current meta-analysis totalled approximately 30 min of PA daily, with a small but statistically significant advantage for PA breaks over continuous exercise. Taken together, the observational and experimental research suggest that PA breaks might have a small advantage over continuous exercise, but any such advantages are abolished with high amounts of daily exercise. However, such comparisons between cross-sectional controlled laboratory studies and observational studies need to be interpreted cautiously, as the results of Ekelund et al. cannot rule out possible effects resulting from patterns of accumulated sitting.

The evidence on the effects of sitting on metabolic health generated in our review is supported modestly by epidemiological evidence. Recent prospective studies of total sitting time and incident type 2 diabetes, in contrast to cross-sectional studies of sedentary time and breaks measured by self-report [117], found little evidence for an association [118], or associations, between sitting behaviour or time and incident type 2 diabetes, but were limited to inactive [119] or obese [120] participants only. To resolve the discordant findings of prospective versus cross-sectional epidemiological studies, which do suggest an association between sitting time and type 2 diabetes [117], future prospective studies utilising accelerometer assessed total sitting time need to be conducted. Few prospective epidemiologic studies to date have assessed the links between breaks and metabolic outcomes, and even fewer support any associations. Baseline breaks, independent of total sitting time, did not predict any metabolic outcomes at 6-month follow-up [121]. Breaks were not associated with all-cause mortality over 5 years of follow up in older men [122]. To our knowledge, only one prospective epidemiological study to date has found an association between longer sedentary behaviour bouts, synonymous with infrequent sedentary breaks, and mortality risk [123]. Sedentary breaks here refer to any break in sedentary behaviour, measured in observational studies typically with Actigraph accelerometry. Cross-sectional studies of breaks that report device-measured sedentary time and breaks also present an unclear picture, with Actigraph measured breaks being inversely associated with some metabolic markers [124, 125]. However, there was little evidence for an association between sedentary breaks, quantified by a thigh worn ActivPAL inclinometer, and diabetes or metabolic syndrome [126]. Conversely, using the same device, number of long sitting bouts was deleteriously associated with several glucose and lipid biomarkers, although somewhat ameliorated by MVPA [127]. Thus, there is conflicting cross-sectional observational evidence, perhaps or perhaps not supporting our findings for a small advantage of PA breaks over continuous exercise. It should be noted that in the current meta-analysis breaks were PA breaks, with standing breaks excluded; thus PA breaks in the included experimental studies are not the same as sedentary breaks in observational studies.

As sedentary behaviour and physical activity guidelines development require some relative consistency between different types of evidence (experimental, epidemiological, etc.), it is surprising how sedentary breaks became part of several national guidelines [5, 128–130] given that only one prospective observational study [123] objectively measured sedentary patterns, and none have used inclinometers, in relation to health risk. Additionally, only 1 experimental study [69] has investigated the effects of the patterning of PA breaks, reporting no differences between PA breaks performed every 20 or 60 min. Recently, the United States of America Physical Activity Guidelines Advisory Committee in its Scientific Report to the Secretary of Health and Human Services [24] concluded that there was insufficient evidence that bouts or breaks in SB are important factors in the relationship between SB and all-cause mortality, and incidence of or mortality from CVD, cancer, or incident type 2 diabetes or weight status. Accumulating brief bouts of PA between bouts of sitting throughout the day in a “whole day” approach [131] might be a feasible alternative for a considerable part of the population who do not exercise, a hypothesis that is supported by the results of the current meta-analysis, which found that there was a small advantage for PA breaks compared to one continuous exercise bout, on glucose, and no difference on insulin and TAG measures, especially as those with higher BMI appeared to benefit more. Therefore, given the results of the current meta-analysis of cross-sectional experimental studies, shedding light on the prospective associations between sedentary breaks and metabolic outcomes is an area of absolute priority for future epidemiological research.

In summary, the results of our systematic review and meta-analysis, viewed in the context of the wider literature, suggest that PA breaks, performed for example, throughout a normal working day, might be an alternative, or at worst, complementary for those who are unable to perform one bout of structured exercise training, particularly in those with higher BMIs, for the prevention of type 2 diabetes and atherosclerosis.

### Future Research, and Reasons for Divergent Results

There was moderate to substantial heterogeneity (Figs. 4, 7) in the results that might be explained by the PA/health status of participants, sex, and also whether a study utilised single or multi-day designs.

It is unclear if the number, duration, intensity, amount and modality of PA breaks within a period of prolonged sitting, and the total duration of the sitting bout, are mediators in the metabolic responses to sitting, with only one study investigating and reporting that such variables did not affect glucose outcomes [69]. Most currently researched modalities involve light to moderate walking or running [7, 8, 13, 20, 21, 46, 68]. To date, only Dempsey et al. [47] and Hawari et al. [78] have examined metabolic responses to simple resistance activities (SRAs) as a means to interrupt sitting. Interestingly, engaging in own body weight resistance type exercises was associated with similar decreased risk of mortality compared to engaging in aerobic type exercise [132]. Pertinently, the modality of the exercise interrupting sitting, walking or cycling at very low intensity, even when energy matched, might play a role in modulating post-prandial glycaemic responses [14]. Future research should attempt to explore the effects of very light intensity breaks, such as fidgeting [133–135], that can be performed at a low enough intensity, or very short duration HIIT [89, 90, 136, 137] breaks in sitting which constitute, “exercise snacks” [138], so as to address concerns about productivity, impracticality, the habitual nature of sitting [139] and management support [140].

Additionally, different sitting periods were used, along with different patterns of PA breaks. Some used 2 day laboratory designs [7, 8, 11, 45], whereas others used 1 day [20, 60, 62, 77]. A free-living protocol over 1 day [70] or 4 days [74, 75] was also used. Participants sat for bouts between 2.5 [16], 4 [77], and 7–9 h [7, 8, 20, 45–47, 60, 62]. Breaking up sitting with exercise might have different effects depending on the duration of sitting, given that observational findings suggest that extended sitting time negatively affects metabolic health [123–125]. However, this is as yet untested experimentally. Additionally, the duration of individual discrete sitting bouts varied, for example 1.5 min of brisk walking every 15 min [20] or every 30 min [62]. Interestingly, when participants had their sleep restricted, PA breaks did not attenuate post-prandial glucose measures compared to sitting. Therefore, future experimental research could systematically explore the effects of the number, duration and intensity of PA breaks, and also the total duration of the sedentary bout in which PA breaks occurred.

People who were overweight [71] or had lower CRF [81] experienced greater attenuation in post-prandial glucose. Subgroup analysis showed that those who were physically inactive, or had IFG or type 2 diabetes, experienced statistically significant greater glycaemic benefits from PA breaks, and attenuation of insulin also approached significance (Figs. 5, 8). In support of this, meta-regression revealed that PA breaks had a greater effect on glucose in participants with higher BMI.

No or small differences in glycaemia or lipaemia between EX and INT were reported in studies involving highly fit young men with maximal aerobic capacity ($\dot{V}$ O_2_max) above 50 ml.kg.min^−1^ [7, 8, 45], whereas studies involving sedentary or metabolically unhealthy people appeared to find in favour of INT for glycaemia [20, 60, 62, 73] or continuous for lipaemia [62]. Glucose transport, uptake and metabolism might be higher in magnitude in the insulin sensitive compared to the insulin resistant [44]. Moreover, trained [42, 43] or insulin sensitive [41] participants demonstrate a greater response to a glucose or lipid challenge. Differences in glycaemia or lipaemia between sitting and PA breaks possibly would be greater in participants who are not exercise trained. Endurance training might alter lipid metabolite levels, composition and localisation, and thus muscle lipid metabolism and insulin sensitivity [41, 141].

We found a small body of evidence suggesting that sex might mediate glucose responses [13, 15, 47, 69] (Figs. 6, 11). Conversely, 2 studies [46, 72] reported no sex interactions for any outcomes when sitting was interrupted by light or moderate walking. Both sex specific PA break protocols or the underlying mechanisms, such as oestrogen levels [48–51], responsible for any possible sex divergent metabolic responses to PA breaks could be avenues for further research. It would be desirable if future studies recruited more than one sex and were powerful enough to analyse and report sex-specific results, even if this was done only for completeness and subsequent findings were put in the appendices.

Meal timings, type of meals, whether high fat [7, 8, 20, 45], high carbohydrate [60], or mixed meals [62, 77, 83], liquid [60, 62] or solid [7, 8, 20, 77, 83], varied. Liquid meals might lower the magnitude of post-prandial excursions [142], thus the results might be affected by whether liquid or solid meals were used. The macronutrient and amino acid composition [143–146], and the glycaemic index (GI) of meals may also modulate post-prandial metabolic responses.

Recently, Bailey et al. [63] reported that the GI of the breakfast meal and PA breaks both independently affected post-prandial glucose excursions, with little evidence that there were additive effects from combining PA breaks with a low GI meal.

Additionally, participants were fed one meal [46, 77] two meals [20], three meals [62] or six small meals [77]. Moreover, participants consumed their own breakfast prior to arrival in the laboratory for exercise trials [7, 8], whereas breakfast was provided for them as part of the test meal in another trial [20]. Therefore, feeding protocols might explain some of the heterogeneity in post-prandial responses, and should be investigated more comprehensively.

Furthermore, blood was drawn, for example, just before PA breaks [46, 47] or in rested, sedentary conditions, 1 day after PA breaks [7, 8], every 10 min [16], once every 2 h [10, 20] or assessed via CGMS [12, 14, 15, 22, 71, 82, 83]. Thus, results could have been affected by differing blood draw protocols [99, 147]. Notably, PA breaks reduced post-prandial iAUC up to 2 h after a meal, but not up to 4 h after [82], suggesting that meal timing in relation to blood draw schedule can significantly affect results.

Few studies so far have attempted to assess the underlying mechanisms responsible for metabolic responses to PA breaks, even if merely by assessing c-peptide, which would determine whether decreases in insulin are the result of decreased insulin secretion or increased clearance [148–151]. Additionally, only a few studies have assessed lipoproteins [77, 79], adipose tissue gene expression [73], molecular signalling involved in glucose metabolism [152], or used new metabolomics methods [153] to assess lipidomics [154], and none have assessed branched-chain amino acids [153].

### Publication Bias

Since visual inspection of funnel plots is subjective and can lead to incorrect interpretations, even by medical researchers [155], a variety of methods were used to assess publication bias [53, 54, 156]. There might have been publication bias, or selective outcome or analysis reporting [56] in TAG measures from comparing PA breaks with sitting especially (Table 3) (Fig. 15). Additionally, there might have been publication bias for insulin measures from PA breaks compared to sitting and PA breaks compared to continuous exercise (Table 3). The Vevea and Woods [58] method estimated that the SMD for TAG measures comparing PA breaks with sitting would be reduced from − 0.27 to − 0.20, assuming severe 2-tailed selection bias (Table 3). Assuming the existence of severe 2-tailed bias, the effects of PA breaks on glucose and insulin would be still moderate, i.e. SMD of − 0.41 and − 0.43, respectively.

### Risk of Bias

No subgroup analysis or meta-regression was performed to assess possible moderating effects of risk of bias on effect size because as stated, only a small number of studies reported randomisation, blinding and handling of data attrition clearly [15, 46, 47]. Future research should more clearly report randomisation, blinding and data attrition procedures, and should also more clearly fully report all data collected, even if statistically non-significant.

### Strength and Weaknesses

The current work has a number of strengths. Experimental controlled studies that evaluated the metabolic effects of PA breaks and those of continuous or prolonged exercise in the context of prolonged sitting were systematically synthesised. The metabolic effects of PA breaks compared to no exercise sitting were also systematically synthesised. A variety of publication bias analyses were conducted, and effect sizes in the event of severe publication bias were also calculated. A meta-regression identified BMI as a moderator for glucose and insulin responses to PA breaks. When data were not reported in a study, they were obtained from the authors.

Despite this, the limitations of the current work must be mentioned. Selection bias is a possibility, as only published peer-reviewed studies were included. The inclusion criteria for the meta-analysis could be a limitation, as only trials with explicitly controlled PA break protocols were included. The exclusion of studies with “free living” protocols might affect the results. However, when free-living trials that did not use strictly controlled laboratory protocols [70, 74–76] were included in the meta-analyses the results and interpretation were not qualitatively substantively altered. For example when comparing EE matched PA breaks with continuous exercise, the SMD for glucose measures would have changed from − 0.26 (95% CI − 0.49 − 0.03, *p *= 0.03) to − 0.23 (95% CI − 0.41, − 0.05, *p *= 0.01). The SMD for insulin measures would have changed from 0.36 (95% CI − 0.45, 1.18, *p *= 0.38) to 0.24 (95% CI − 0.37, 0.84, *p *= 0.44), whereas the SMD for TAG would have changed from 0.06 (95% CI − 0.23, 0.36, *p *= 0.67) to − 0.01 (95% CI − 0.27, 0.26, *p *= 0.96). Similarly, when PA breaks were compared with sitting, with free-living trials included, the SMD for glucose measures changed from − 0.54 (95% CI − 0.70, − 0.37, *p *= 0.00001) to − 0.51 (95% CI − 0.67, − 0.35, *p *= 0.00001), for insulin measures from − 0.56 (95% CI − 0.74, − 0.38, *p *= 0.00001) to − 0.54 (95% CI:− 0.71, − 0.38, *p *= 0.00001), and for TAG measures from − 0.26 (95% CI − 0.44, − 0.09, *p *= 0.004) to − 0.31 (95% CI − 0.48, − 0.15, *p *= 0.0002).

In studies with more than 2 experimental conditions, the sample size of the control condition, uninterrupted sitting was divided by the number of times it was used as a control. For example, Pulsford et al. [21] had 3 experimental conditions, walking, cycling, and sitting. Therefore, in the meta-analyses, the sample size for the sitting condition was divided in half, as the sitting condition was used twice as the control comparison. When the results of experimental PA breaks conditions were combined instead, the SMD for glucose measures, comparing PA breaks with sitting, changed from − 0.54 (95% CI − 0.70, − 0.37, *p *= 0.00001) to − 0.52 (95% CI − 0.70, − 0.35, *p *= 0.00001). The SMD for insulin measures changed from − 0.56 (95% CI − 0.74, − 0.38, *p *= 0.00001) to − 0.54 (95% CI − 0.73, − 0.35, *p *= 0.00001) and the SMD for TAG measures changed from − 0.26 (95% CI − 0.44, − 0.09, *p *= 0.004) to − 0.26 (95% CI − 0.44, − 0.09, *p* = 0.005). The SMD for glucose measures, comparing PA breaks with continuous exercise, changed from − 0.26 (95% CI − 0.49, − 0.03, *p *= 0.03) to − 0.26 (95% CI − 0.50, − 0.02, *p *= 0.04).

Furthermore, the scales used to measure metabolic responses—CGMS, iAUC, tAUC—were heterogeneous. One study collected both CGMS [15] and venous blood [47] measurements. The current meta-analysis utilised the CGMS data from Dempsey et al. [15]. If the venous blood data from Dempsey et al. [47] were utilised instead, the SMD for blood glucose, for PA breaks versus sitting, would have changed from − 0.54 (95% CI − 0.70, − 0.38) to − 0.50 (95% CI − 0.65, − 0.35).

Altenburg et al. [67] was omitted from the meta-analysis, as data were not normally distributed and reported in medians, but inclusion would likely not have altered the main results. The meta-analysis included one study that was not randomised [77]. However, removing it would not have affected the results. TAG SMD for INT compared to SIT changed from − 0.27 (95% CI − 0.45, − 0.08, *p *= 0.005) to − 0.28 (95% CI − 0.48, − 0.08, *p *= 0.005). Similarly, TAG for INT compared to EX changed from 0.04 (95% CI − 0.23, 0.31, *p *= 0.77) to 0.00 (95% CI − 0.30, 0.29, *p *= 0.98).

Studies that included only interrupted sitting with standing were not included, as standing might not exceed 1.5 METs [29, 30] and heterogeneity in EE during standing might be affected by leg or body displacement [30]. Additionally, normal weight men and women, BMI: 22.5 ± 1.5 kg/m^2^, had higher leg muscle activity during sitting compared to the overweight, BMI: 28.4 ± 2.9 kg/m^2^. Conversely, leg muscle activity was higher in overweight adults during standing [31]. However, standing might confer positive [157] or negative [158, 159] physiological effects beyond simply EE, and thus a future meta-analysis should evaluate the effects of using standing to break up sitting.

Only BMI was assessed as a moderator variable in the meta-regression. $\dot{V}{\text{O}}_{2\text{max} }$ could not be evaluated, as generally only studies with aerobically fit or physically active participants reported $\dot{V}{\text{O}}_{2\text{max} }$ or $\dot{V}{\text{O}}_{{2{\text{peak}}}}$ values [7, 8, 11, 16, 45, 62]. Future experimental work should attempt to assess CRF, as it has been suggested that CRF might modulate responses to PA breaks in sitting [160]. Similarly, exercise intensity could not be assessed in the meta-regression due to intensity being reported either as absolute or relative intensity. Nor could the prior PA levels of participants be assessed as a continuous moderator because some studies merely reported highly aerobically fit participants as “recreationally active” [7, 8] whereas others did not report PA status [63, 68, 77]. Similarly, this meant that in the subgroup analyses (Figs. 3, 5, Electronic Supplementary Material Appendix S2—Figs S3, S5 and S6), studies were grouped such that participants in one subgroup were physically active, or sedentary, or were overweight/obese or had type 2 diabetes or IFG, compared to another subgroup with active people. It should be noted that differences observed in subgroup analyses (Fig. 15) based on summary data are considered observational, and need to be specifically tested in within subjects experimental designs, for example in participants with lower compared to higher BMI. Similarly, even though BMI was identified as a moderator in the meta-regression, this was based on summary data, is observational [86] and needs to be specifically tested in future experimental studies. Similarly, effect sizes were calculated using summary data from individual studies, and not individual participant level data.

## Conclusion

Interrupting sitting with PA attenuates post-prandial glucose, insulin, and TAG, with greater glycaemic attenuation in people with high BMI. There was a small benefit for PA breaks compared to one continuous bout of exercise on glucose measures when exercise protocols were energy matched, and the difference was practically non-existent for insulin and TAG. The effect sizes were similar to those observed in meta-analyses of various traditional exercise protocols in diverse populations. Assuming that the acute metabolic effects we detected translate into long term metabolic benefits, PA breaks might be an alternative or adjunct to a single structured aerobic exercise bout, or more specifically, structured walking, running, or cycling, in people with higher BMI.

## Electronic supplementary material

Below is the link to the electronic supplementary material.

Supplementary material 1 (DOCX 11 kb)

Forest plot together with risk of bias assessment for the effects of PA breaks vs no-exercise sitting on TAG, stratified by sex (TIFF 992 kb)

Forest plot for the effects of physical activity breaks vs sitting on insulin, stratified by sex (TIFF 1133 kb)

Forest plot for the effects of physical activity breaks vs continuous exercise on triacylglyerol, active vs inactive/unfit/T2D/IFG (TIFF 831 kb)

Forest plot for the effects of PA breaks vs continuous exercise on TAG, stratified by sex (TIFF 772 kb)

Forest plot for the effects of physical activity breaks vs continuous exercise on glucose, active vs inactive/unfit/T2D/IFG (TIFF 1053 kb)

Forest plot for the effects of physical activity breaks vs continuous exercise on insulin, active vs inactive/unfit/T2D/IFG (TIFF 906 kb)

Forest plot for the effects of PA breaks vs continuous exercise on insulin, stratified by sex (TIFF 759 kb)
