# Supplementary material for: Effects of Interrupting Prolonged Sitting with Physical Activity Breaks on Blood Glucose, Insulin and Triacylglycerol Measures: A Systematic Review and Meta-analysis
Source: Sports Med. 2019 Sep 24;50(2):295–330. doi: 10.1007/s40279-019-01183-w (PMC6985064; doi:10.1007/s40279-019-01183-w)
Supplement: Supplementary file 1 — Supplementary material 1 (DOCX 11 kb) [file 40279_2019_1183_MOESM1_ESM.docx]

**Electronic Supplementary Material Appendix S1. Search terms and deduplication procedure**

Search terms, for PubMed and SportDiscus: ("sedentary behaviour" OR "sedentary behavior" OR "prolonged sitting" OR "sitting" OR "rested") AND ("exercise" OR "walking" OR "standing" OR "physical activity") AND ("breaks" OR "intervals" OR "breaking" OR "break" OR "intermittent" OR "interrupting" OR "short bouts" OR "alternating") AND ("glucose" OR "insulin" OR "glycaemia" OR "glycemia" OR "lipids" OR "triglycerides" OR "lipaemia" OR "lipemia" OR "energy expenditure" OR "blood pressure" OR "lipoprotein" OR "metabolic" OR "metabolic" OR "mortality" OR "diabetes" OR "cholesterol" OR "LDL" OR "HDL").

Search terms, Science Direct: tak(("sedentary behaviour" OR "sedentary behavior" OR "prolonged sitting" OR "sitting" OR "rested") AND ("exercise" OR "walking" OR "standing" OR "physical activity") AND ("breaks" OR "intervals" OR "breaking" OR "break" OR "intermittent" OR "interrupting" OR "short bouts" OR "alternating") AND ("glucose" OR "insulin" OR "glycaemia" OR "glycemia" OR "lipids" OR "triglycerides" OR "lipaemia" OR "lipemia" OR "energy expenditure" OR "blood pressure" OR "lipoprotein" OR "metabolic" OR "metabolic" OR "mortality" OR "diabetes" OR "cholesterol" OR "LDL" OR "HDL") )

Search terms, OvidSP: (("sedentary behaviour" or "sedentary behavior" or "prolonged sitting" or "sitting" or "rested") and ("exercise" or "walking" or "standing" or "physical activity") and ("breaks" or "intervals" or "breaking" or "break" or "intermittent" or "interrupting" or "short bouts" or "alternating") and ("glucose" or "insulin" or "glycaemia" or "glycemia" or "lipids" or "triglycerides" or "lipaemia" or "lipemia" or "energy expenditure" or "blood pressure" or "lipoprotein" or "metabolic" or "metabolic" or "mortality" or "diabetes" or "cholesterol" or "LDL" or "HDL")).ab,ct,hw,id,kw,mh,nt,ot,sh,ti.

Deduplication

Subsequently, all files were copied into a common folder for deduplication. Duplicates were removed using the Find Duplicates->Close Duplicates in this Folder option, with automatically identified duplicates manually checked before removal. Subsequently, the Find Duplicates->Exact Duplicates option was used, with manual checking again performed prior to removal. Lastly, any duplicates that persisted were manually removed during study selection process.
