# Supplementary figures and images for: Effects of Interrupting Prolonged Sitting with Physical Activity Breaks on Blood Glucose, Insulin and Triacylglycerol Measures: A Systematic Review and Meta-analysis
Source: Sports Med. 2019 Sep 24;50(2):295–330. doi: 10.1007/s40279-019-01183-w (PMC6985064; doi:10.1007/s40279-019-01183-w)

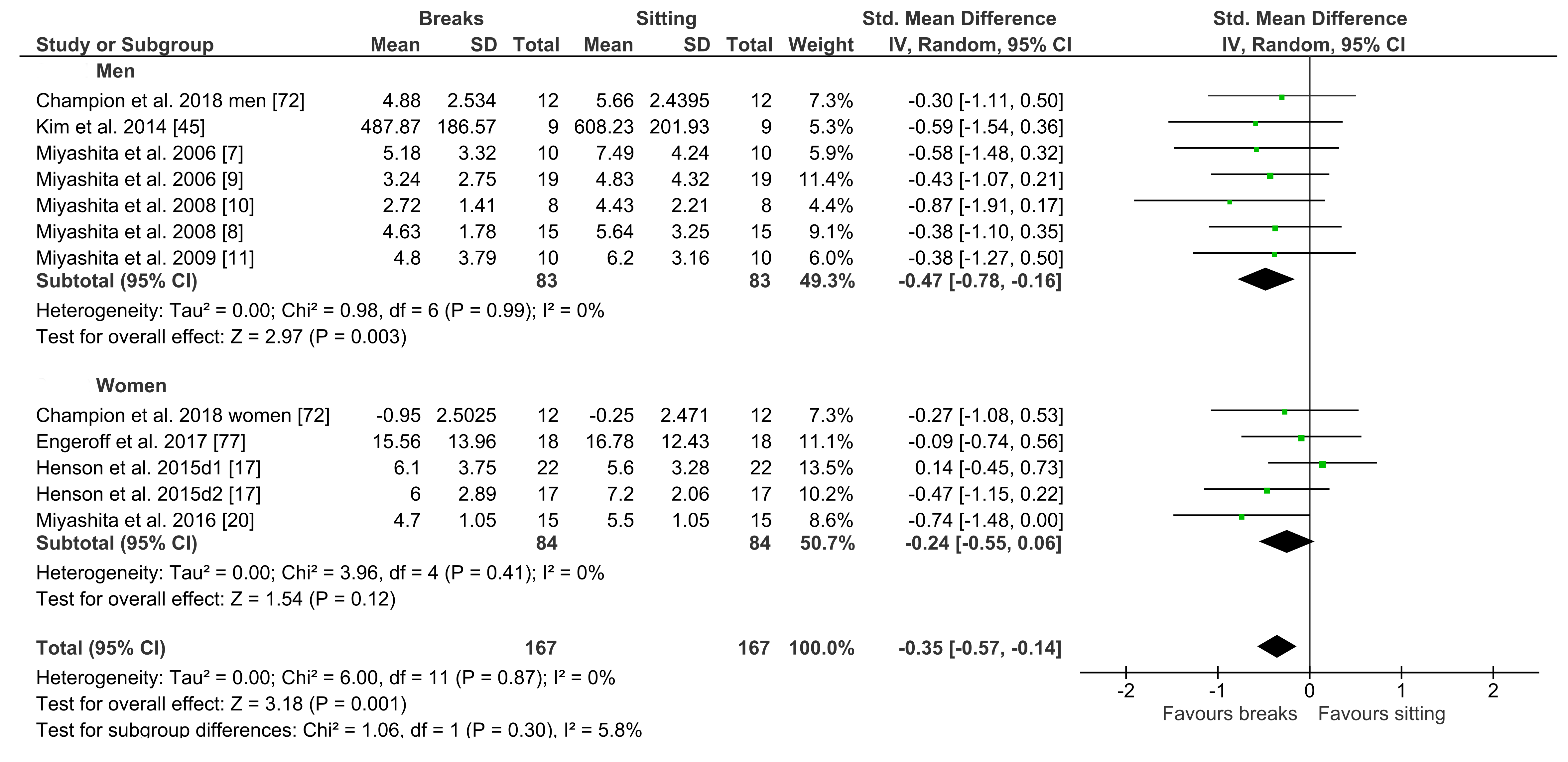

Supplement: Supplementary file 2 — Forest plot together with risk of bias assessment for the effects of PA breaks vs no-exercise sitting on TAG, stratified by sex (TIFF 992 kb) [file 40279_2019_1183_MOESM2_ESM.tif]

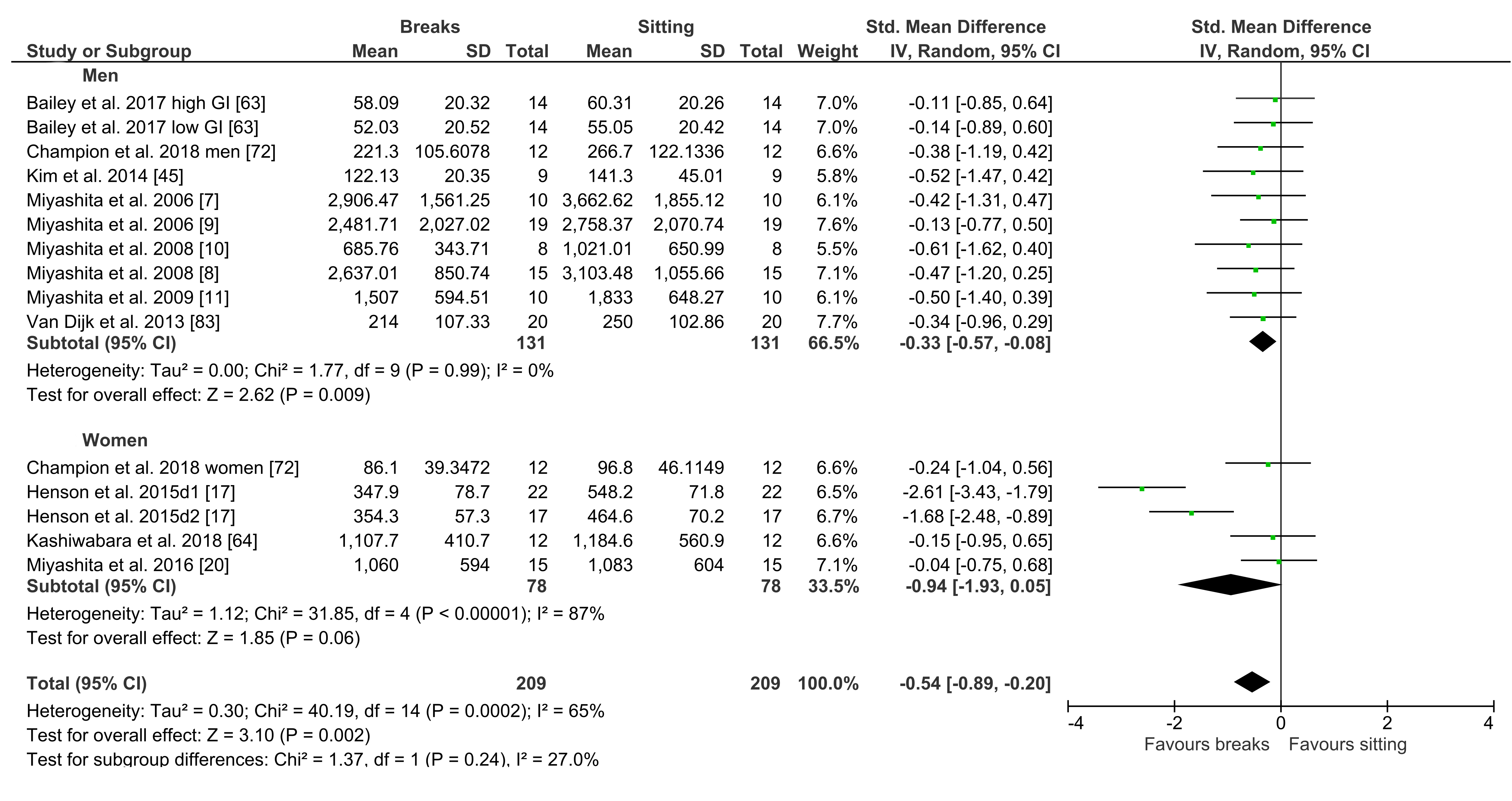

Supplement: Supplementary file 3 — Forest plot for the effects of physical activity breaks vs sitting on insulin, stratified by sex (TIFF 1133 kb) [file 40279_2019_1183_MOESM3_ESM.tif]

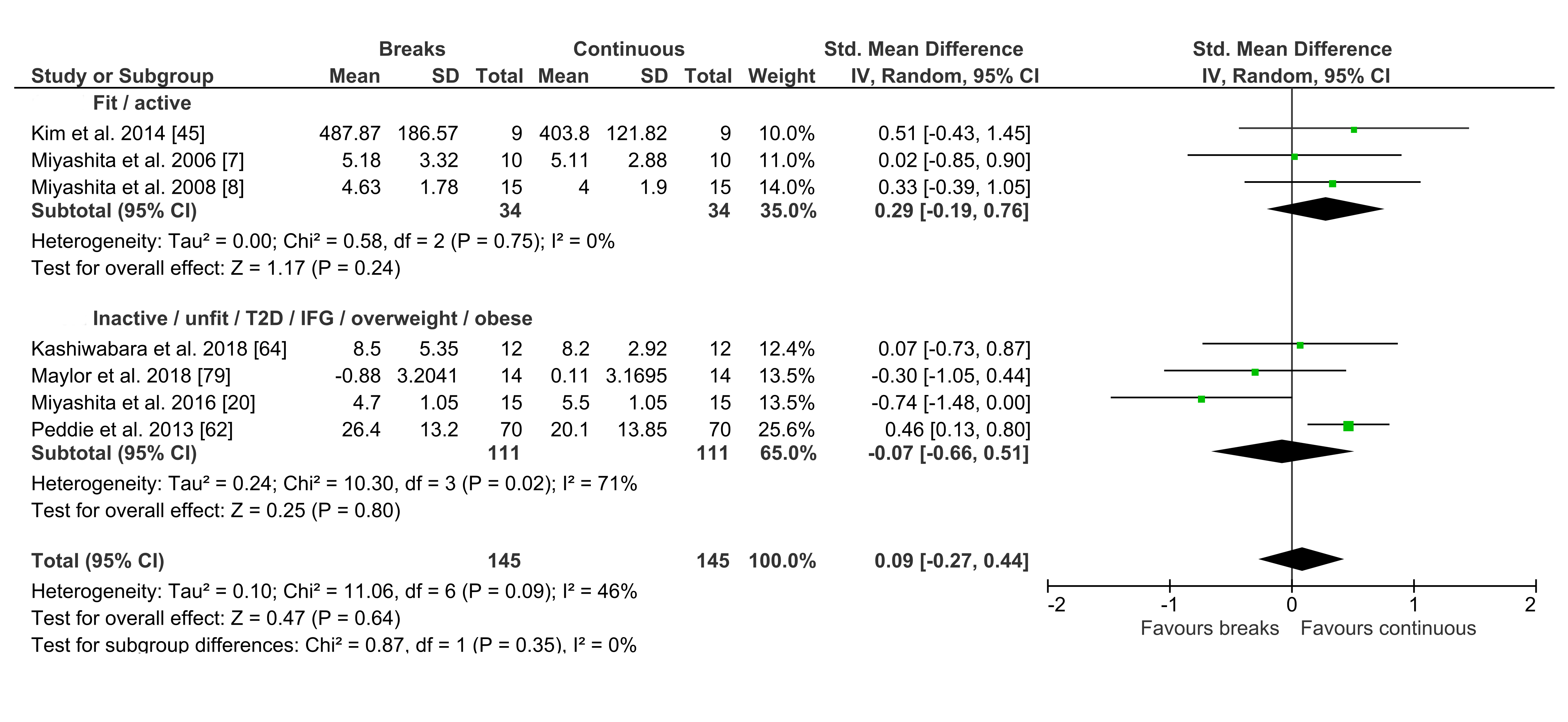

Supplement: Supplementary file 4 — Forest plot for the effects of physical activity breaks vs continuous exercise on triacylglyerol, active vs inactive/unfit/T2D/IFG (TIFF 831 kb) [file 40279_2019_1183_MOESM4_ESM.tif]

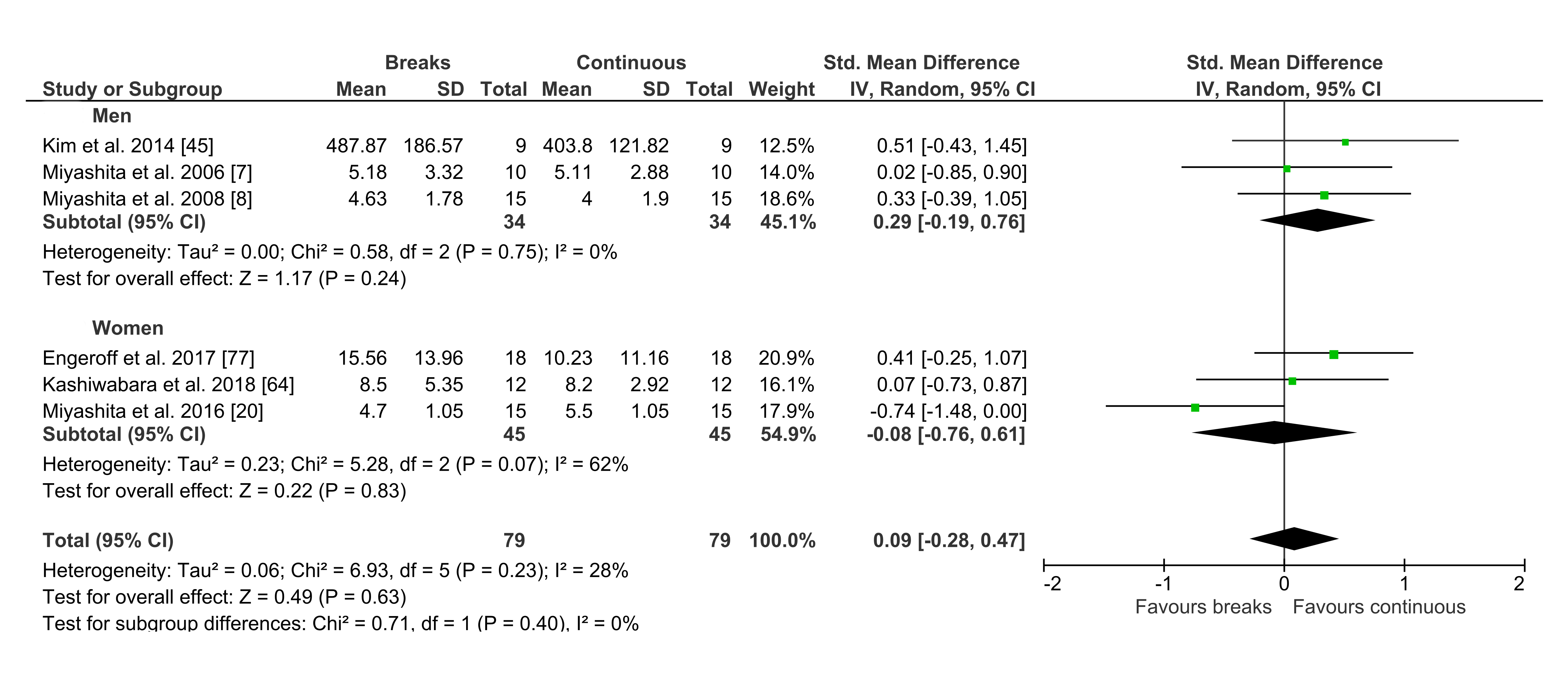

Supplement: Supplementary file 5 — Forest plot for the effects of PA breaks vs continuous exercise on TAG, stratified by sex (TIFF 772 kb) [file 40279_2019_1183_MOESM5_ESM.tif]

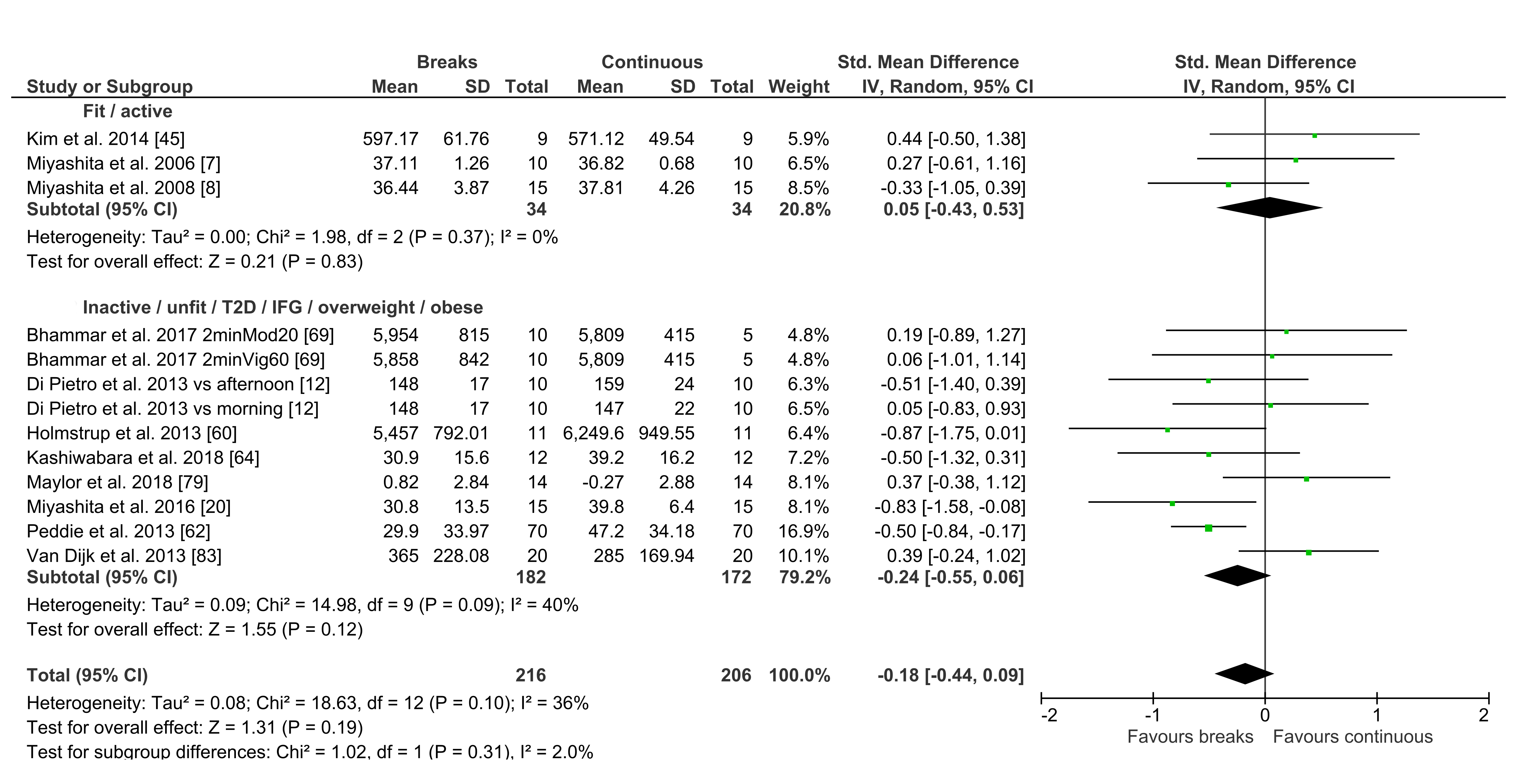

Supplement: Supplementary file 6 — Forest plot for the effects of physical activity breaks vs continuous exercise on glucose, active vs inactive/unfit/T2D/IFG (TIFF 1053 kb) [file 40279_2019_1183_MOESM6_ESM.tif]

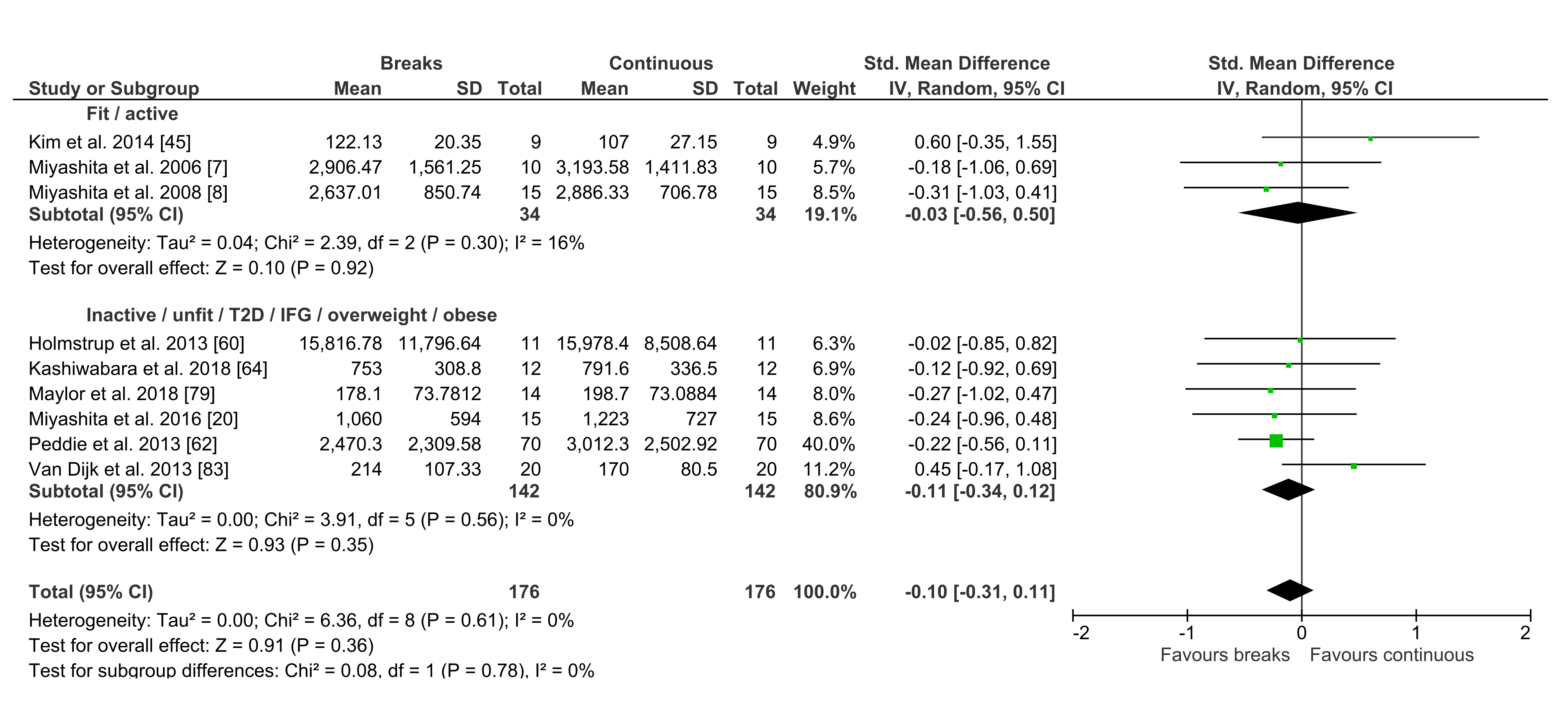

Supplement: Supplementary file 7 — Forest plot for the effects of physical activity breaks vs continuous exercise on insulin, active vs inactive/unfit/T2D/IFG (TIFF 906 kb) [file 40279_2019_1183_MOESM7_ESM.tif]

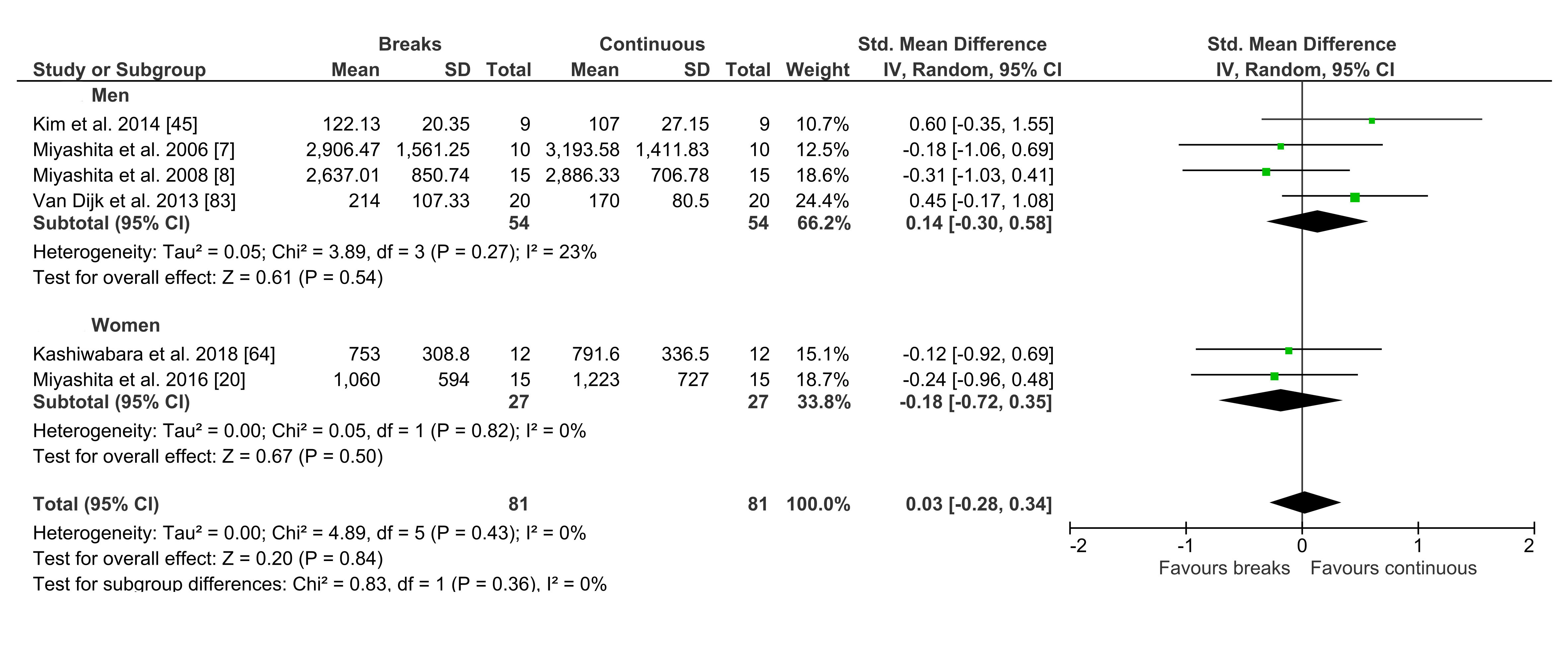

Supplement: Supplementary file 8 — Forest plot for the effects of PA breaks vs continuous exercise on insulin, stratified by sex (TIFF 759 kb) [file 40279_2019_1183_MOESM8_ESM.tif]
